# Genome wide association mapping of agro-morphological traits among a diverse collection of finger millet (*Eleusine coracana* L.) genotypes using SNP markers

**DOI:** 10.1371/journal.pone.0199444

**Published:** 2018-08-09

**Authors:** Divya Sharma, Apoorv Tiwari, Salej Sood, Gautam Jamra, N. K. Singh, Prabina Kumar Meher, Anil Kumar

**Affiliations:** 1 Department of Molecular Biology & Genetic Engineering, G.B. Pant Univ. of Agriculture and Technology, Pantnagar, Uttarakhand, India; 2 Sam Higginbottom University of Agriculture, Technology & Sciences, Allahabad, Uttar Pradesh, India; 3 ICAR-Vivekananda Institute of Hill Agriculture, Almora, Uttarakhand, India; 4 Department of Genetics & Plant Breeding, College of Agriculture, G.B. Pant Univ. of Agriculture & Technology, Pantnagar, Uttarakhand, India; 5 Division of Statistical Genetics, ICAR-Indian Agricultural Statistics Research Institute, New Delhi, India; National Institute of Plant Genome Research, INDIA

## Abstract

Finger millet (*Eleusine coracana* L.) is an important dry-land cereal in Asia and Africa because of its ability to provide assured harvest under extreme dry conditions and excellent nutritional properties. However, the genetic improvement of the crop is lacking in the absence of suitable genomic resources for reliable genotype-phenotype associations. Keeping this in view, a diverse global finger millet germplasm collection of 113 accessions was evaluated for 14 agro-morphological characters in two environments viz. ICAR-Vivekananda Institute of Hill Agriculture, Almora (E1) and Crop Research Centre (CRC), GBPUA&T, Pantnagar (E2), India. Principal component analysis and cluster analysis of phenotypic data separated the Indian and exotic accessions into two separate groups. Previously generated SNPs through genotyping by sequencing (GBS) were used for association mapping to identify reliable marker(s) linked to grain yield and its component traits. The marker trait associations were determined using single locus single trait (SLST), multi-locus mixed model (MLMM) and multi-trait mixed model (MTMM) approaches. SLST led to the identification of 20 marker-trait associations (MTAs) (p value<0.01 and <0.001) for 5 traits. While advanced models, MLMM and MTMM resulted in additional 36 and 53 MTAs, respectively. Nine MTAs were common out of total 109 associations in all the three mapping approaches (SLST, MLMM and MTMM). Among these nine SNPs, five SNP sequences showed homology to candidate genes of *Oryza sativa* (Rice) and *Setaria italica* (Foxtail millet), which play an important role in flowering, maturity and grain yield. In addition, 67 and 14 epistatic interactions were identified for 10 and 7 traits at E1 and E2 locations, respectively. Hence, the 109 novel SNPs associated with important agro-morphological traits, reported for the first time in this study could be precisely utilized in finger millet genetic improvement after validation.

## Introduction

Finger millet [*Eleusine coracana* (L.) Gaertn] is a potential future crop because of its adaptability to environmental fluctuations and high nutritional value. It is a self-pollinating allotetraploid (AABB) with basic chromosome number of 9 (2n = 4x = 36). The crop is thought to be domesticated in Uganda about 5000 years ago from where its cultivation extended to the Ethiopian highlands and India. Among millets, finger millet ranks fourth in importance after sorghum, pearl millet and foxtail millet. The area occupied by the crop at the global level is estimated to be approximately 4.0–4.5 million hectares with a production of 5 million tonnes annually [1]. The high nutritive value of finger millet coupled with its ability to thrive under poor soil fertility and low rainfall conditions make it a climate-smart crop.

Improvement to a crop generally involves exploiting the existing genetic variability in desired traits, and nature as well as degree of their association among them [2]. Most of the agro-morphological characters are controlled by multiple genes showing complex inheritance. Genetic mapping of functional loci associated with important agronomical traits facilitate their easy manipulation in crop improvement programmes using linked markers. Linkage analysis and association mapping are the two most widely used techniques for dissecting complex and polygenic traits [3]. Grain yield is a complex trait and is considered to be a definitive product of its component traits. Therefore, it is necessary to have knowledge on the extent of association between grain yield and its contributing characters. The selection of superior accessions based on grain yield alone is difficult due to the integrated make-up of plant in which most of the traits are interrelated and being governed by a number of genes. Hence, finding genetic and environmental controls of grain yield and its components would allow breeders to efficiently manipulate these traits. Manipulation of these traits would potentially raise the current yield levels in finger millet which at present are very low (500 to 2000 kg/ha). Moreover, this approach has the potential to deliver nutritionally superior genotypes of the finger millet which primarily fulfils the goals of the World Health Organization (WHO) to alleviate malnutrition among populations in developing countries globally.

Association mapping is considered to be one of the most promising tools for plant breeders. It is based on linkage disequilibrium (LD) and overcomes the drawbacks of classical linkage analysis which is based on biparental mapping populations. Moreover, this approach helps in the detection of a large number of alleles and enhances the mapping resolution [4,5]. However, single locus single trait (SLST), the most widely used association mapping approach for genome-wide association studies (GWAS) is also not free from bias [6]. Therefore, advanced models viz., multi-locus mixed model (MLMM) and multi-trait mixed model (MTMM) approaches are recently introduced to address the issues of background noise and pleiotropy [7,8]. MLMM takes care of background noise similar to composite interval mapping (CIM) [7], while MTMM allows detection of individual QTLs that are pleiotropic [8]. In most of the GWAS studies epistasis is never considered but has been taken care of in the present study.

The availability of SSR markers in finger millet is limited [9,10] and only a few reports are available on association mapping using SSR markers [11–13]. The SNPs are considered to be the ideal markers in plant genetic studies and genome analyses because of their excellent genetic attributes, such as ubiquitous distribution, co-dominant nature of inheritance, chromosome-specific location and high reproducibility [14–19]. SNP mining becomes highly challenging in allo-tetraploid crops such as finger millet due to low polymorphism level resulting from high degree of inbreeding and large numbers of homeologous SNPs, which are the result of polymorphism between the AA and BB sub-genomes of the same individual [10]. But, identification of SNPs in several other polyploid crops including wheat [20], cotton [21], oats [22] and groundnut [23]has been successful as a result of suitable filtering tools and stringency in mapping parameters [24]. The genotyping by sequencing (GBS) data has the potential to provide large number of SNPs which could help in determining marker trait associations to facilitate marker based strategies for genetic manipulations to introduce improved finger millet genotypes [25]. Hence, in the present study, association of grain yield and its component traits with SNPs was investigated to understand the genetic basis of grain yield and its components in a diverse gene pool. This information would enable us to identify favourable alleles that could be introgressed and stacked into elite accessions as a strategy to raise the yield of finger millet crop. This is the first report of association mapping of important agro-morphological traits of finger millet using SNPs.

## Materials and methods

### Experimental materials and evaluation sites

The material comprises 113 diverse finger millet accessions representing world collections from India, Nepal, Africa, Maldives and Germany. Of the 113 accessions, 56 (IE numbers) belong to International Crops Research Institute for the Semi-Arid Tropics (ICRISAT) mini core collection, 23 accessions (GE numbers) belong to All India Coordinated Small Millets Improvement project core collection, 16 accessions (VHC lines) are landraces from Uttarakhand hills and 18 others are improved genotypes including white grain genotypes of Indian National Agricultural Research System (NARS). The distribution of selected finger millet accessions across the world has been discussed by Kumar et al. [25]. The field trials were conducted at two locations *viz*. ICAR-Vivekananda Institute of Hill Agriculture, Almora (Hawalbag) experimental farm (E1) (25.35° N latitude and 79.39° E longitude, 1250 m above msl, mean rainfall-1000mm) and Crop Research Centre (CRC), GBPUA&T, Pantnagar (E2) (29° N latitude and 79.38° E longitude, 243.84 m above msl, mean rainfall-1434mm) in the year 2015. These two locations represent two different ecosystems of finger millet cultivation *i*.*e*. hill ecosystem and plain ecosystem.

### Experimental design and data collection

The crop was raised in an alpha lattice experimental design in two replications as described by Barreto et al. [26]during the rainy season. The plants were raised in 3m long rows spaced 22.5cm apart. The plot size was three rows of each genotype in both the locations. Extra plants were removed within a month of planting to maintain plant to plant spacing of 10cm. All the recommended package and practices were followed to raise a healthy crop. However, no disease management was practised in the crop. Observations were recorded on five randomly chosen plants for fourteen quantitative characters following finger millet descriptors (IBPGR, 1985). The list of agro-morphological characters along with their description is given below (Table 1).

**Table 1:** List of agro-morphological traits consisting of 14 quantitative characters studied among finger millet accessions.

S.No.	Trait	Code	Character Description of Traits
1.	Days to 50% flowering Days to Flowers	DF	From sowing to stage when ears have emerged from 50% of main tillers
2	Days to maturity	DM	From sowing to stage when 50% of main tillers have mature ears.
3	Basal tiller number	BT	Number of basal tillers which bears mature ears
4	Plant height (cm)	PH	From the ground level to tip of inflorescence at dough stage
5	Culm Thickness (cm)	CT	Diameter of internode between third and fourth node from top at dough stage
6	Flag leaf blade length (cm)	FLBL	Measured from ligule to leaf tip at flowering
7	Flag leaf blade width (cm)	FLBW	Measured across centre of flag leaf at flowering
8	Peduncle length (cm)	PL	From top most node to base of the thumb finger
9	Ear length (cm)	EL	From base tip of inflorescence to top of inflorescence at dough stage
10	Ear width (cm)	EW	Measured across centre of the inflorescence at dough stage
11	Length of longest finger (cm)	LLF	From the base to tip of longest spike on main tiller at dough stage
12	Width of longest finger (cm)	WLF	Measured across centre of longest finger at dough stage
13	Fingers number per ear	FN	The number of fingers present in the main ear head at dough stage was counted
14	Grain Yield (g/plot)	GY	Grain yield per plot

### Statistical analysis

Analysis of variance (ANOVA) for alpha lattice design was done in Indian NARS statistical computing portal of ICAR-IASRI, New Delhi using SAS software (SAS 9.3) for each location separately. The adjusted mean values of all agro-morphological traits for both the locations were used for further analyses. JMP 9.0 (JMP, Version 9.0.0. SAS Institute Inc., Cary, NC) was used for hierarchical cluster and principal component analyses, while correlation analysis was done using R software [27]. Heritability was worked out as per Johnson et al. [28].

### Linkage disequilibrium

The SNP data generated earlier by Kumar et al. [25] was used for linkage disequilibrium (LD). The level of LD was calculated using the TASSEL v.5.2 software for each marker pair together with the significance of the parameter. LD was estimated for the 113 finger millet genotypes by computing the squared correlation coefficient (r^2^). The analysis was conducted with consideration of the admixed genotype identified by STRUCTURE at K = 3. The LD-values between all pairs of loci were plotted as triangle LD plots to estimate the general view of genome-wide LD patterns using TASSEL software.

### Marker-trait association (MTA) analysis

Single locus single trait association mapping was performed using TASSEL version 5.2 (http://www.maizegenetics.net), which involves association of individual markers/locus with each of the 14 agro-morphological traits, employing the two models- General Linear Model (GLM) and Mixed Linear Model (MLM) based on the kinship matrix (K model). The kinship matrix was generated in TASSEL by converting the distance matrix obtained from TASSEL’s cladogram function into similarity matrix. P < 0.01 was set as the significant threshold for the association analysis.

Additionally, GWAS was also conducted using MTMM [8] and MLMM [7]. For MLMM, background genome was considered as a cofactor (like CIM in interval mapping) using stepwise mixed-model regression with forward inclusion and backward elimination, and was performed by using R-version of MLMM (available at https://cynin.gmi.oeaw.ac.at/home/resources/mlmm) [7]. For MTMM, all pairs of traits showing significant and strong correlation (p < 0.05; r^2^ > 0.2) were used (S2 Table). Three different tests viz. (i) full test- for comparing the full model including the effect of a marker accession and its interactions with the model that included neither, (ii) interaction effect test-for comparison of the full model to one which does not include interactions, and (iii) common effect test- for comparing a model with a marker accession to the model that does not include marker accession [8] were performed in MTMM.. False discovery rate (FDR) was used for calculating the critical p-values for each trait separately, which was found to be highly stringent to determine the significance of marker-trait associations (MTAs).

Marker-trait associations detected through SLST, MTMM and MLMM, were subjected to two dimensional epistatic interaction analyses for individual trait. This analysis was performed using the function interaction “Pval” available in SNPassoc package [29] of R-software [27].

### In-silico comparative genomics

In-silico comparative genomics analysis was conducted for the common marker sequences of identified QTLs which were found to be linked with the agronomic traits in all the three approaches. Due to the unavailability of finger millet reference genome, cross species validation of SNP markers was done to see if there is any sequence similarity using Basic Local Alignment Search Tool (BLAST). The sequences of monocot plants viz., rice (*Oryza sativa*), wheat (*Triticum aestivum*), maize (*Zea mays*), foxtail millet (*Setaria italica*), sorghum (*Sorghum bicolor*) and purple false brome or Switchgrass (*Panicum virgatum*) were used for BLAST (nucleotide) search. This was carried out using Phytozome v10.3 (http://phytozome.jgi.doe.gov/pz/portal.html), an online web tool [30]. The regions of sequence similarity found on the chromosome of the model plants were analyzed for the presence of candidate gene(s) near the QTL sequences. The functions of associated candidate gene(s) were further analyzed for their relatedness to useful agronomic traits. The Kyoto Encyclopedia of Genes and Genomes (KEGG) pathway database was used to identify the biological pathways involved in finger millet grain yield.

## Results

### Variation for agro-morphological traits

**Fig 1:**
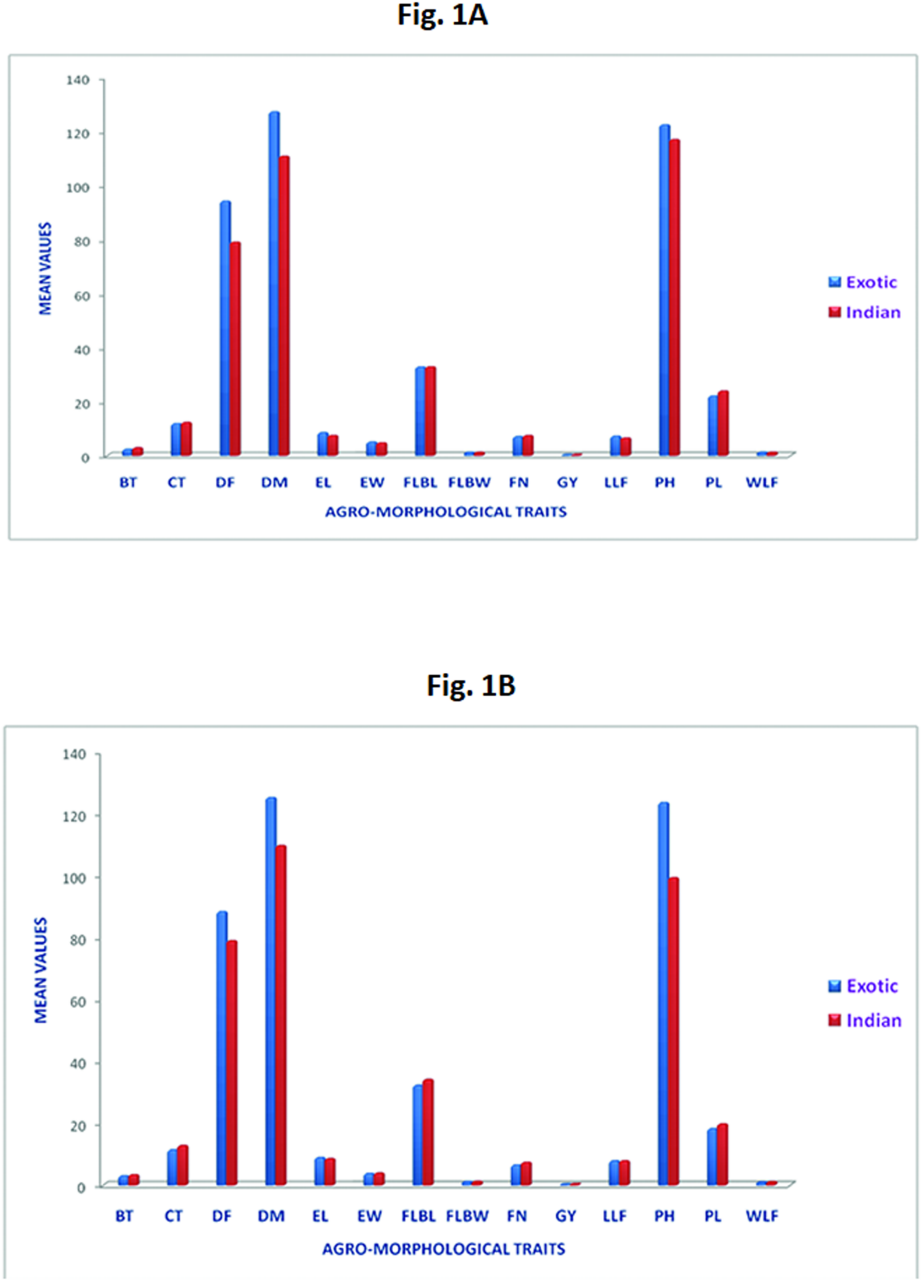
A)Comparison of Indian and exotic finger millet accessions of E1 location (Almora) for agro-morphological traits under study (BT- Basal tiller number, CT- Culm Thickness, DF- Days to 50% flowering, DM- Days to 50% maturity, EL- Ear length, EW- Ear width, FLBL- Flag leaf blade length, FLBW- Flag leaf blade width, FN- Fingers per head, GY- Grain yield, LLF- Length of longest finger, PH- Plant height, PL- Peduncle length, WLF- Width of longest finger). B) Comparison of Indian and exotic finger millet accessions of E2 location (Pantnagar) for agro-morphological traits under study (BT- Basal tiller number, CT- Culm thickness, DF- Days to 50% flowering, DM- Days to 50% maturity, EL- Ear length, EW- Ear width, FLBL- Flag leaf blade length, FLBW- Flag leaf blade width, FN- Fingers per head, GY- Grain yield, LLF- Length of longest finger, PH- Plant height, PL- Peduncle length, WLF- Width of longest finger).

Analysis of variance for both the locations viz. ICAR-Vivekananda Institute of Hill Agriculture, Almora (Hawalbag) experimental farm (E1) and Crop Research Centre (CRC), GBPUA&T, Pantnagar (E2), resulted in highly significant differences (P<0.01) among accessions for most of the traits, except for PH, FLBW at E2 and WLF at E1(S1 Table). We observed a wide range of variation in the agronomic performance of the accessions (Table 2). The highest grain yield per plot was found in the accession IE 3618 in both the environments (520 g in E1 and 540g in E2) followed by GPU 26 (440 g in E1 and 470 g in E2), IE 2710 (420 g in E1 and 430 g in E2), IE 2043 (410 g in E1 and 450 g in E2) and IE 2296 (400 g in E1 and 440 g in E2). The traits BT, CT, PH and FLBL observed wide range of variation at E2 in comparison to E1, while the range of variation for DM, FN and GY was almost similar in both the locations. The overall trend for all the observed traits however was similar in both the locations. Thus, all these traits could be considered as good candidates for marker-trait associations (Table 2). It was observed from the phenotypic data that accessions from the Indian sub-continent were smaller in height and took less number of days for flowering and maturity across the locations in comparison to the accessions representing African continent (Fig 1A and 1B). Heritability in broad sense estimates of the fourteen quantitative traits ranged from 24.7% for WLF to 93.9% for DM at E1 and 33.6% for EL to 92.3% for FLBL at E2 (Table 2). Heritability was classified as low (below 30%), medium (30–60%) and high (above 60%) as suggested by Johnson et al. [28]. Considering this delineation, high heritability values were observed for DM (93.9%) followed by DF (87.6%), LLF (79.1%), EL (75.8%), EW (67.1%), GY (64.5%), FN (64.3%) and PH (64.5%) at E1 and PH (91.4%), FLBW (92.3%), GY (89.4%), DM (86.6%), CT (79.7%), FN (78.5%), PL (72.5%), WLF (71.3%), LLF (70%) and BT (69.7%) at E2. Rest of the traits in their respective locations observed medium to low heritability.

**Table 2:** Range observed for 14 agro-morphological traits at E1 (Hilly region) and E2 (Plain region).

S.No.	Trait	Range		Standard Deviation (SD)		Heritability (h^2^%)	
		E1	E2	E1	E2	E1	E2
1	**PH (**cm**)**	79.96–147.40	38.82–189.08	13.75	20.53	64.5	91.4
2	**CT (**cm**)**	0.81–1.57	0.24–2.32	1.23	0.42	32.3	79.7
3	**BT** (number)	1–5	1–11	0.83	1.75	32.3	69.7
4	**FLBL (**cm**)**	20.89–44.82	15.33–56.66	4.17	7.09	54.1	92.3
5	**FLBW (**cm**)**	0.7–1.31	0.62–2.13	0.07	0.25	38.5	52.2
6	**PL (**cm**)**	13.56–31.26	6.52–28.11	3.24	4.73	60.4	72.5
7	**DF** (days)	55–124	59–110	14.06	10.65	87.6	39.2
8	**DM** (days)	89–147	87–146	13.99	13.17	93.9	86.6
9	**EL (**cm**)**	4.34–19.62	2.45–16.09	2.00	2.46	75.8	33.6
10	**EW (**cm**)**	2.62–11.12	0.98–7.06	0.96	1.22	67.1	50.1
11	**FN** (number)	5–11	4–12	1.20	1.43	64.3	78.5
12	**LLF (**cm**)**	4–16	2.81–14.42	1.69	1.85	79.1	70
13	**WLF** (cm)	1.0–3.44	0.4–2.14	0.22	0.31	24.7	71.3
14	**GY** (g)	0.0–520	0.0–540	0.09	0.10	64.5	89.4

**Fig 2:**
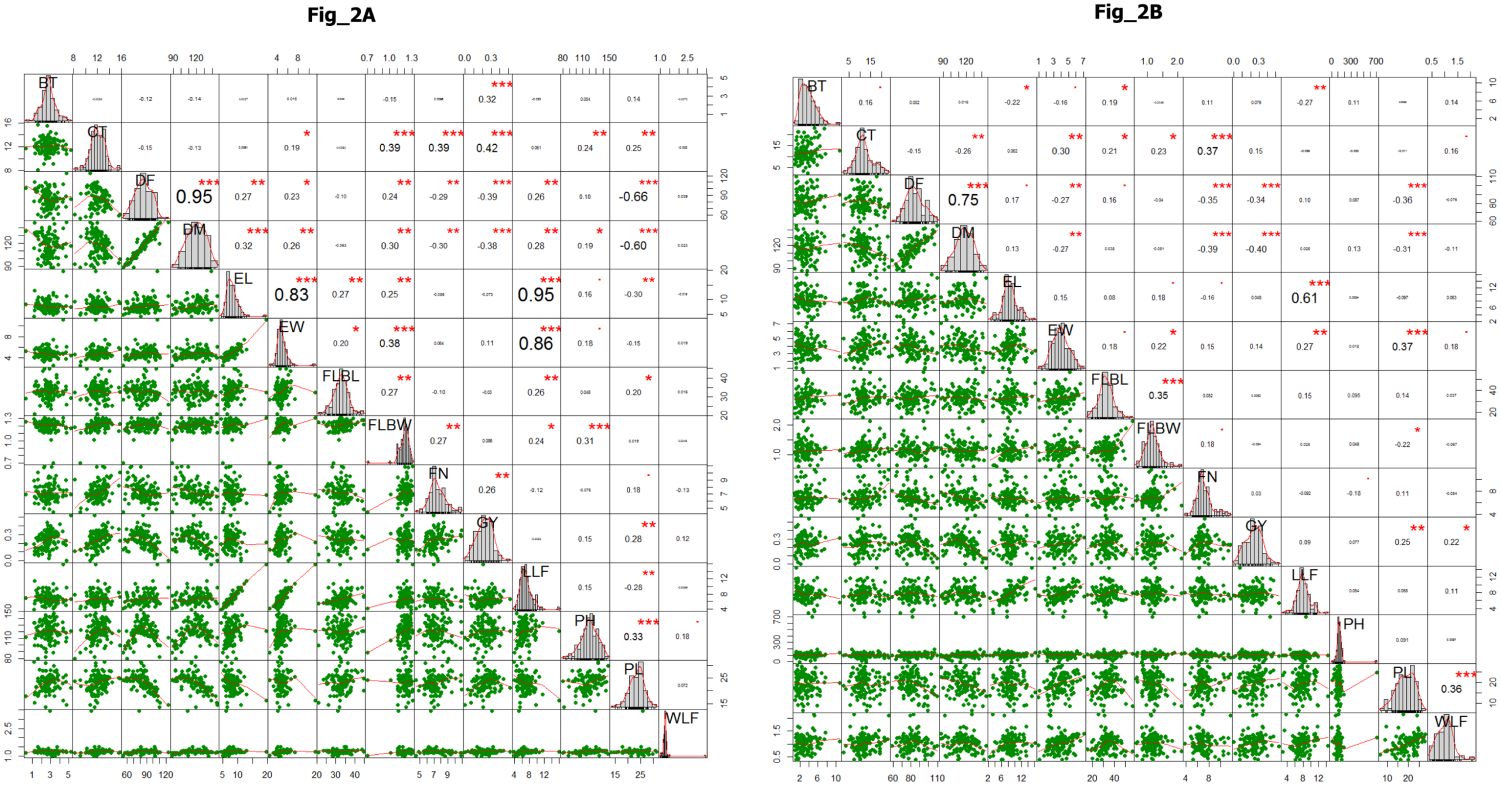
A) Correlation coefficients (above diagonal) and linear regressions (below diagonal) among 14 agro-morphological traits (abbreviations and units, see Table 1), based on 113 accessions of finger millet at E1 location (Almora). *, **, *** Significant at 0.05, 0.01, and 0.001 probability levels, respectively. B) Correlation coefficients (above diagonal) and linear regressions (below diagonal) among 14 agro-morphological traits (abbreviations and units, see Table 1), based on 113 accessions of finger millet at E2 location (Pantnagar). *, **, *** Significant at 0.05, 0.01, and 0.001 probability levels, respectively.

Phenotypic association analysis showed positive significant correlation of DF with DM, EL, EW, FLBW and LLF in E1 (Fig 2A). Grain yield showed significant correlation with FN, CT and BT. Positive and significant correlation was also found between EL, LLF and EW. However, EW was negatively correlated with DF in E2. Significant and positive correlation was found between EL and LLF at E2. Highly significant and positive correlations were observed between DF and DM across the two locations (0.95 at E1 and 0.75 at E2). Moreover, highly significant and negative correlation was observed for GY with both DF and DM at both the locations (E1 and E2) (Fig 2A and 2B).

### Principal component and cluster analysis

**Fig 3:**
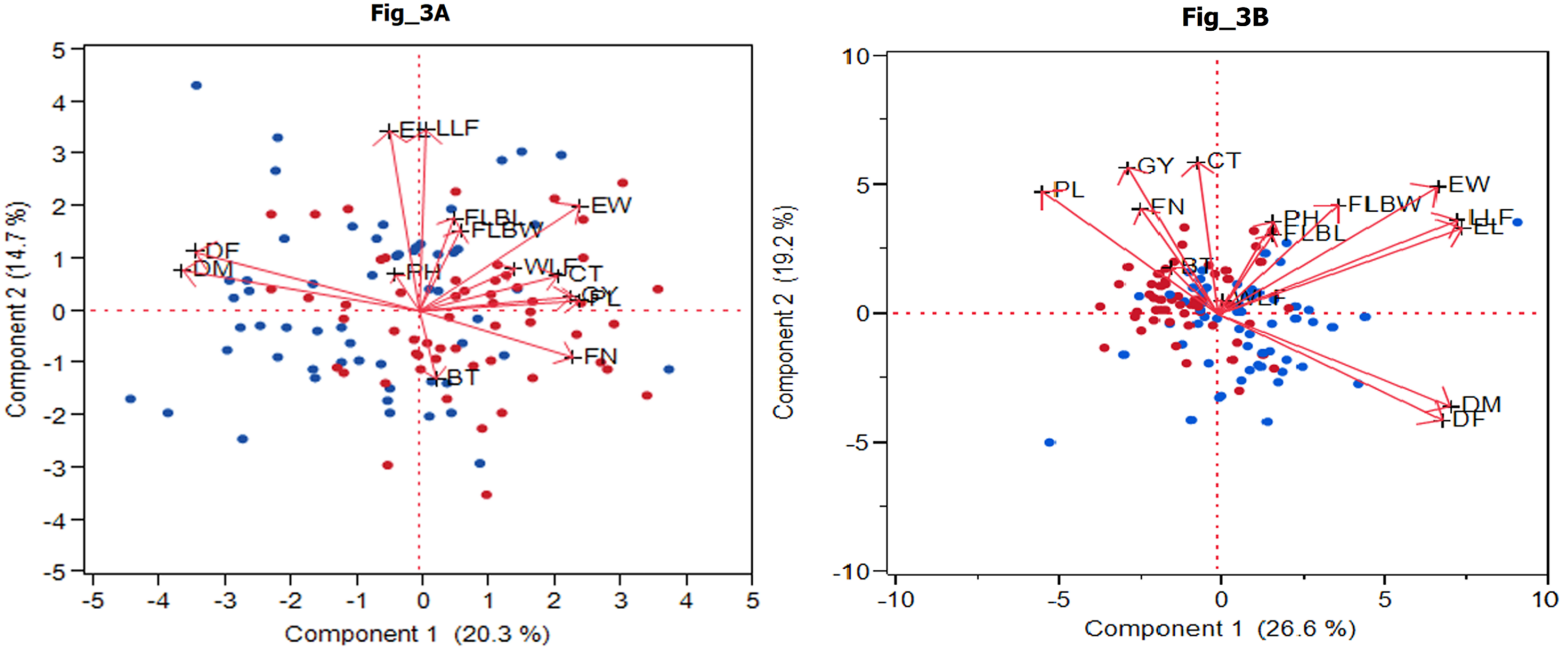
A) Two-dimensional plot showing the variability of 14 traits across 113 accessions of finger millet at E1 location (Almora). B) Two-dimensional plot showing the variability of 14 traits across 113 accessions of finger millet at E2 location (Pantnagar).

Principal component analysis (PCA) was used to evaluate interrelationships among different traits. The first five PCA components provided a reasonable summary of the data and explained 75 and 65 per cent of the total variation in E1 and E2 locations, respectively. The first principal component (PC1) was the most important and explained 26 and 20 per cent of the total variation in E1 and E2 locations, respectively (Fig 3A and 3B). PC1 was attributed to EL, LLF, DM, DF and EW for largest positive loadings at E1, whereas, PL, EW, FN, GY, CT and WLF had largest positive loadings at E2. PL, GY and FN had largest negative loadings at E1. The second PC explained 19 and 15 percent of the total variation at E1 and E2, respectively. The variation was attributed to positive loadings of CT, GY, EW and PL at E1; and LLF, EL and EW at E2. The third PC, which explained 11 and 12 per cent of the total variation at E1 and E2, respectively, differentiated the accessions by FLBW, CT, DM, DF and PH at E1, and FLBW, CT, FLBL and FN at E2. The parameters closer to grain yield in the biplot were positively correlated with grain yield (FN, BT, CT and PL at E1; PL, CT, FN and WLF at E2) and those away from grain yield forming greater angle i.e. DF and DM were weakly or negatively associated in both the locations (Fig 3A and 3B).

Two-way cluster analysis separated the accessions into two major groups in both the locations (S1a and S1b Fig). Group A contained 81 and 84 accessions at E1 and E2, respectively, all of them were mostly of Indian origin with few exceptions. This group was subdivided into 9 and 8 subgroups at E1 and E2, respectively. Group B contained 32 and 29 accessions at E1 and E2, respectively, mostly belonging to exotic lines of African origin. This group was further subdivided into 3 and 4 subgroups at E1 and E2, respectively.

### Linkage disequilibrium

The linkage disequilibrium (LD) of the studied genotypes was determined using SNP markers. In total 17.9% (r^2^ ≥0.05) and 2.9% (r^2^ ≥ 0.1) of the marker pairs based on r^2^ estimates showed significant LD. Triangle plots for pairwise LD between SNP markers demonstrated significant LD blocks in the genome-wide LD analysis (S2 Fig).

### Marker-trait association (MTA) using single locus single trait (SLST) analysis

Marker-trait association analysis for 14 quantitative traits with GBS data (Kumar et al., 2016), resulted in the identification of 499 linked SNPs through GLM, whereas, MLM showed 303 associated SNPs for 14 traits at a significant threshold level (P<0.01; P<0.001) in E1 location. Two hundred and sixty-one (261) marker-trait associations were found common in both GLM and MLM analysis for 14 quantitative traits at E1 location. In E2 location, 433 linked SNP were identified through GLM, while MLM analysis showed 308 SNP-trait associations for 14 traits at a significant threshold level (P<0.01; P<0.001). Both GLM and MLM had 242 SNPs in common for fourteen agro-morphological traits at E2. Association analysis also revealed many multiple SNP-trait associations and single SNP-multiple trait associations in both the locations i.e. in several cases one or more SNPs were significantly associated with more than one trait and vice versa (S3 Table).

The marker-trait association analysis results showed twenty common SNP-trait associations in both the environments (E1 & E2) through GLM and MLM approaches (Table 3). Out of these 20 associations, SNPs TP1071491, TP133182, TP1600637 were associated with DF and explained phenotypic variance of 9.5%, 11.4%, 19.4%, respectively at E1 and 10.6%, 14.8% and 18.5%, respectively at E2. The SNP TP351642 was associated with EL with R^2^ value of 9.5% at E1 and 10.2% at E2. GY was found to be associated with eight SNPs *viz*., TP1071491, TP1310091, TP1431319, TP1510146, TP28411, TP64954, TP760377 and TP878687, which showed phenotypic variance of 12.4%, 10.1%, 15.2%, 10.7%, 16.8%, 14.3%, 15.0% and 10.7%, respectively at E1 and 11.1%, 11.1%, 16.4%, 10.1%, 16.4%, 14.7%, 13.4% and 10.2%, respectively at E2. The next trait with higher number of trait-SNPs associations was DM, which was associated with seven SNPs viz., TP133182, TP1381676, TP1600637, TP214730, TP414254, TP627932 and TP872087, explaining phenotypic variance of 12.2%, 12.4%, 25.4%, 20.6%, 18.2%, 30.3%, 11.4%, respectively at E1 and 9.1%, 16.8%, 25.7%, 19.8%, 16.5%, 26.8%, 9.45%, respectively at E2. GY and DF were associated with a common SNP, TP1071491. Similarly, DF and DM also showed association with a common SNP, TP1600637. FLBL was associated with a SNP (TP641195) explaining phenotypic variance of 9.9% and 11.8% at E1 and E2, respectively (Table 3).

**Table 3:** The details of genomic SNP markers associated with agronomic traits using GLM and MLM approach in 113 accessions of finger millet.

Marker QTL	Trait	GLM (P<0.01)				MLM (p<0.01)			
		p-value		R^2^ (%)		p-value		R^2^ (%)	
		Hilly region (E1)	Plain region (E2)	Hilly Region (E1)	Plain region (E2)	Hilly region (E1)	Plain region (E2)	Hilly region (E1)	Plain region (E2)
TP1071491	DF	9.82E-03	1.47E-03	8.55%	11.90%	8.17E-03	5.20E-03	9.48%	10.60%
TP133182	DF	2.06E-03	5.56E-04	11.00%	11.90%	3.03E-03	6.66E-04	11.40%	14.80%
TP1600637	DF	1.25E-03	1.09E-03	19.40%	11.90%	2.60E-03	3.98E-03	19.40%	18.50%
TP133182	DM	1.41E-03	8.79E-03	11.70%	11.90%	1.94E-03	9.30E-03	12.20%	9.12%
TP1381676	DM	4.15E-03	7.62E-04	14.60%	11.90%	8.45E-03	1.69E-03	12.40%	16.80%
TP1600637	DM	7.05E-05	5.90E-05	26.30%	11.90%	4.38E-04	5.19E-04	25.40%	25.70%
TP214730	DM	1.54E-04	2.42E-04	22.70%	11.90%	1.36E-03	1.92E-03	20.60%	19.80%
TP414254	DM	1.84E-03	3.44E-03	18.70%	11.90%	2.38E-03	7.04E-03	18.20%	16.50%
TP627932	DM	1.01E-04	3.45E-04	31.10%	11.90%	1.84E-04	4.26E-04	30.30%	26.80%
TP872087	DM	1.66E-03	6.26E-03	11.60%	11.90%	3.00E-03	8.22E-03	11.40%	9.45%
TP351642	EL	9.28E-03	1.84E-03	8.58%	11.90%	8.13E-03	3.87E-03	9.51%	10.20%
TP641195	FLBL	3.13E-03	4.67E-03	10.50%	11.90%	6.81E-03	2.65E-03	9.92%	11.80%
TP1071491	GY	1.43E-03	1.25E-03	12.10%	11.90%	6.21E-03	4.33E-03	10.40%	11.10%
TP1310091	GY	3.04E-03	3.97E-03	10.50%	11.90%	2.63E-03	4.02E-03	11.90%	11.10%
TP1431319	GY	1.89E-03	5.38E-03	17.80%	11.90%	3.51E-03	5.06E-03	19.40%	16.40%
TP1510146	GY	3.56E-03	2.85E-03	10.20%	11.90%	6.33E-03	6.30E-03	10.00%	10.10%
TP28411	GY	2.22E-03	3.35E-03	17.80%	11.90%	3.46E-03	5.60E-03	17.60%	16.40%
TP64954	GY	5.58E-03	5.75E-03	14.40%	11.90%	8.00E-03	5.05E-03	14.10%	14.70%
TP760377	GY	8.40E-05	4.57E-03	24.80%	11.90%	1.19E-03	8.03E-03	20.70%	13.40%
TP878687	GY	4.70E-03	2.90E-03	9.77%	11.90%	9.26E-03	6.06E-03	9.25%	10.20%

### Marker-trait association using MLMM (Multi-Locus Mixed Model Approach)

Fifty-three marker-trait associations were identified for eight agro-morphological traits (DF, DM, EL, FLBL, FN, GY, LLF and PH) at E1 and E2 using MLMM approach (Table 4). Ten of the 53 marker-trait associations were common with those identified through SLST approach. These 10 MTAs were TP133182 for DF, TP214730 and TP872087 for DM, TP1071491, TP28411 TP1510146, TP878687, TP1310091, TP1431319 and TP760377 for GY.

**Table 4:** Significant marker-trait associations (MTAs) identified using MLMM (Multi-Locus Mixed Model).

Trait	Marker	P value	
		E1	E2
**BT**	TP959938	1.54E-03	1.54E-03
**DF**	TP1085280	5.01E-03	5.01E-03
	TP133182	2.97E-04	2.97E-04
	TP214730	7.86E-03	7.86E-03
	TP12628	2.35E-03	2.35E-03
	TP1084458	4.99E-03	4.99E-03
	TP404137	2.25E-03	2.25E-03
	TP1578768	8.36E-03	8.36E-03
	TP1414942	1.63E-03	1.63E-03
	TP1071491	4.41E-03	4.41E-03
	TP1242062	7.83E-03	7.83E-03
	TP698175	2.54E-03	2.54E-03
	TP1086421	4.61E-03	4.61E-03
	TP28411	2.02E-03	2.02E-03
	TP1329370	5.09E-03	5.09E-03
	TP473053	7.86E-03	7.86E-03
	TP103874	1.27E-03	1.27E-03
**DM**	TP28411	4.61E-03	4.61E-03
	TP872087	2.74E-04	2.74E-04
	TP979901	9.07E-03	9.07E-03
	TP692180	3.02E-04	3.02E-04
	TP1565065	6.95E-03	6.95E-03
	TP1581043	2.54E-04	2.54E-04
	TP1521889	4.61E-03	4.61E-03
	TP290701	6.86E-03	6.86E-03
**EL**	TP107921	3.44E-03	3.44E-03
**FLBL**	TP18663	9.05E-04	9.05E-04
**FN**	TP1377207	3.47E-03	3.47E-03
	TP9600	7.96E-04	7.96E-04
**GY**	TP1510146	1.28E-03	1.28E-03
	TP1439022	1.55E-03	1.55E-03
	TP735980	7.48E-03	7.48E-03
	TP1310258	8.85E-03	8.85E-03
	TP752454	4.69E-03	4.69E-03
	TP1001349	5.06E-03	5.06E-03
	TP486645	1.14E-03	1.14E-03
	TP878687	1.57E-03	1.57E-03
	TP560402	2.79E-03	2.79E-03
	TP642659	2.79E-03	2.79E-03
	TP1310091	4.58E-03	4.58E-03
	TP962050	2.74E-03	2.74E-03
	TP28411	1.69E-03	1.69E-03
	TP741818	4.07E-03	4.07E-03
	TP45157	4.38E-03	4.38E-03
	TP103874	6.07E-04	6.07E-04
	TP498100	1.10E-03	1.10E-03
	TP1355465	9.94E-03	9.94E-03
	TP1431319	4.90E-03	4.90E-03
	TP1003465	9.92E-04	9.92E-04
	TP595219	8.81E-03	8.81E-03
	TP40780	8.67E-03	8.67E-03
	TP760377	1.57E-03	1.57E-03
**LLF**	TP1062143	2.72E-03	2.72E-03

### Marker-trait association using MTMM (Multi-Trait Mixed Model Approach)

Seventy common marker-trait associations were identified through MTMM analysis using full test at E1 and E2 representing 12 pairs of correlated traits. (Table 5). Further, interaction test led to the identification of 46 associations and common test identified 24 associations for both the locations. Twenty-four pleiotropic QTLs were identified using common test: DF-DM (6), DF-EW (2), DF-GY (1), DM-EW (2), DM-GY (1), EL-LLF (6), GY-PL (5) and CT-FN (1). There were two pairs of correlated traits, namely, DM-GY and DM-PL for which a maximum of 9 and 10 MTAs were found (Table 5). On the contrary, for the following two pair of traits, namely, EW-FLBL, and CT-FN, a solitary MTA was found. Of the 70 associations in MTMM, seven MTAs were common with SLST, sixteen MTAs were common with MLMM analyses and 9 MTAs *viz*., TP1071491, TP133182, TP214730, TP872087, TP1310091, TP1431319, TP28411, TP760377 and TP878687 were common among all the three approaches (Tables 3–5).

**Table 5:** Significant marker-trait associations (MTAs) identified using MTMM.

Trait Combination	Marker	P Value					
		E1			E2		
		Full test	Common	Specific	Full test	Common	Specific
**DF/DM**	TP1071491	2.77E-04	5.07E-05	-	7.93E-04	1.77E-04	-
	TP1085280	1.62E-04	2.90E-05	-	9.81E-07	1.50E-07	-
	TP1193783	1.44E-06	2.11E-07	-	1.35E-04	2.73E-05	-
	TP1234781	6.47E-05	1.10E-05	-	2.67E-04	6.21E-05	-
	TP12628	7.03E-05	1.20E-05	-	2.60E-06	3.89E-07	-
	TP133182	3.60E-06	5.49E-07	-	7.58E-06	1.56E-06	-
**DF/EW**	TP1071491	3.66E-03	1.09E-03	-	5.63E-03	2.29E-03	-
	TP12628	8.33E-03	-	8.83E-03	1.36E-04	-	3.70E-05
	TP963414	1.69E-03	4.02E-04	-	9.69E-03	2.79E-03	-
**DF/FN**	TP1193783	1.03E-03	-	5.88E-04	1.76E-03	-	0.000376
	TP12628	2.10E-03	-	6.79E-04	4.99E-05	-	1.05E-05
	TP133182	4.59E-04	-	2.48E-04	3.23E-05	-	8.21E-06
	TP1377207	2.49E-03	-	7.19E-04	3.23E-03	-	0.000791
	TP1414942	1.01E-03	-	2.92E-04	7.10E-04	-	0.000171
**DF/GY**	TP103874	1.49E-05	-	2.51E-06	6.66E-04	-	0.000134
	TP1051370	9.45E-03	-	2.35E-03	2.38E-03	-	0.000556
	TP1071491	4.47E-03	-	1.47E-03	3.77E-03	-	0.001256
	TP1085280	2.31E-03	-	5.77E-04	1.87E-05	-	4.27E-06
	TP1193783	4.26E-04	-	1.47E-04	4.45E-03	-	0.001029
	TP1003465	5.86E-04	1.71E-04	-	1.51E-03	3.23E-04	-
**DF/PL**	TP1071491	3.90E-04	-	7.58E-05	5.58E-03	-	0.002296
	TP1084351	2.83E-04	-	6.08E-05	9.36E-03	-	0.002276
	TP1084436	8.80E-04	-	1.80E-04	9.29E-03	-	0.002281
	TP1267216	1.68E-03	-	5.38E-04	2.36E-03	-	0.000805
	TP1298228	6.32E-03	-	1.81E-03	4.32E-03	-	0.000982
	TP133182	1.27E-04	-	3.66E-05	4.52E-04	-	0.000523
	TP1349783	7.40E-03	-	1.82E-03	1.79E-04	-	0.000356
	TP1414942	1.80E-04	-	3.53E-05	2.12E-03	-	0.000749
**DM/EW**	TP12628	6.46E-03	-	8.06E-03	1.19E-04	-	3.92E-05
	TP1574043	5.02E-03	1.21E-03	-	2.07E-03	9.84E-03	-
	TP1193783	6.85E-04	3.12E-03	-	2.90E-03	1.78E-03	-
**DM/FN**	TP1193783	2.49E-04	-	2.22E-04	9.43E-05	-	2.29E-05
	TP1234781	3.69E-03	-	2.14E-03	6.04E-04	-	0.000271
	TP12628	1.62E-03	-	5.77E-04	4.38E-05	-	1.06E-05
	TP133182	8.24E-04	-	4.28E-04	4.52E-04	-	0.000101
	TP214730	6.56E-03	-	5.66E-03	2.06E-03	-	0.001095
	TP531558	3.91E-03	-	6.33E-03	5.48E-03	-	0.003807
	TP698175	6.54E-03	-	4.65E-03	6.63E-03	-	0.008532
	TP701554	2.24E-03	-	1.31E-03	3.48E-03	-	0.004814
**DM/GY**	TP103874	9.12E-05	-	1.85E-05	4.30E-05	-	0.000219
	TP1003465	1.50E-03	7.67E-04	-	6.71E-03	2.54E-03	-
	TP1084759	1.36E-03	-	1.73E-03	1.06E-03	-	0.001705
	TP1085280	1.03E-03	-	2.88E-04	3.22E-03	-	1.04E-05
	TP1193783	1.02E-04	-	4.94E-05	2.44E-04	-	7.87E-05
	TP752454	1.98E-03	-	4.69E-04	1.58E-03	-	0.000443
	TP760377	1.22E-03	-	3.64E-04	8.81E-03	-	0.00249
	TP878687	2.89E-04	-	5.94E-05	2.96E-04	-	5.71E-05
	TP962050	1.29E-03	-	3.18E-04	5.99E-03	-	0.002251
**DM/PL**	TP1084436	1.49E-03	-	3.20E-04	3.23E-03	-	0.00075
	TP1084759	1.28E-04	-	3.54E-05	6.45E-03	-	0.007011
	TP1084844	7.23E-04	-	1.58E-04	9.46E-03	-	0.003965
	TP1085280	6.84E-03	-	7.08E-03	9.32E-04	-	0.001128
	TP122612	2.94E-03	-	6.66E-04	2.88E-03	-	0.001482
	TP1234781	1.66E-06	-	3.00E-07	8.67E-04	-	0.000485
	TP12628	8.20E-04	-	2.24E-04	1.41E-03	-	0.003374
	TP133182	2.29E-04	-	6.53E-05	5.94E-03	-	0.004181
	TP1355538	9.26E-03	-	2.49E-03	4.08E-03	-	0.00542
	TP1414942	1.22E-04	-	2.48E-05	3.38E-04	-	0.000209
**EL/LLF**	TP1329979	4.32E-04	8.20E-05	-	2.55E-03	2.24E-03	-
	TP1513250	5.74E-03	1.31E-03	-	2.01E-03	7.85E-03	-
	TP334661	4.50E-03	1.00E-03	-	8.90E-03	1.58E-03	-
	TP351642	2.11E-03	4.41E-04	-	1.86E-04	2.18E-03	-
	TP806011	4.81E-04	9.12E-05	-	4.96E-03	1.12E-03	-
	TP808138	1.72E-03	3.57E-04	-	7.91E-03	1.97E-03	-
**GY/PL**	TP1298228	7.27E-03	5.23E-05	-	6.37E-03	9.47E-03	-
	TP1431319	7.44E-04	2.15E-03	-	9.55E-03	1.25E-03	-
	TP150396	2.27E-04	1.48E-04	-	2.80E-03	4.34E-03	-
	TP498100	7.08E-04	2.12E-04	-	1.86E-03	4.20E-04	-
	TP878687	3.22E-03	1.84E-03	-	1.44E-03	4.06E-04	-
**CT/FN**	TP200121	6.19E-03	1.47E-03	-	9.18E-03	2.26E-03	-

Note: MTMM was performed for pairs of significantly correlated traits (p ≤ 0.05; r^2^ ≥ 0.2)

### Marker-trait associations involved in epistatic interactions

Around 67 epistatic interactions were identified for 10 traits (out of 14 traits) at E1 location (S4 Table). Similarly, 14 epistatic interactions were identified for 7 traits (out of 14 traits) in E2 location. A maximum of 17 interactions were observed for EW and a minimum of one interaction was identified for FN at E1. Similarly, a maximum of three interactions were observed for EW and LLF and a minimum of one interaction was identified for EL and PL at E2 location (S4 Table).

### *In silico* comparative genomics

Nine SNPs which were found common in all the three MTA approaches (SLST, MLMM and MTMM) in both the environments were subjected to local alignment (BLASTx) with the genomes of monocot plants. Orthologues of 8 of the 9 common SNPs were present in the genomes of maize (*Zea mays*), rice (*Oryza sativa*), foxtail millet (*Setaria italica*), wheat (*Triticum aestivum*), sorghum (*Sorghum bicolor*) and purple false brome or Switchgrass (*Panicum virgatum*). The SNPs, TP1071491, TP133182, TP214730, TP1310091, TP872087, TP28411, TP760377 and TP1431319 showed multiple hits, while the SNP TP878687 showed no hit in the genomes of monocot species (Table 6). The KEGG pathway database which helps in systematic understanding of the molecular interactions among genes, in terms of networks/ pathways [31], led to the identification of two major pathways viz. photosynthetic and oxidative phosphorylation pathways which might be involved in finger millet grain yeild enhancement(S3 Fig).

**Table 6:** The details of SNP markers associated with candidate genes of *Oryza sativa* and *Setaria italica* analyzed by *in silico* comparative genomics.

Marker	Associated trait	Name of the species	Chromosome/scaffold position in respective species	Locus name	Name of the gene	Function
TP1071491	DF	*Setaria italica*	Scaffold 14	Seita.J020700.1	ATP synthase subunit beta, mitochondrial	Increases rate of photosynthetic electron transport, Co2 assimilation and plant growth
TP214730	DM	*Oryza sativa*	Chr10	LOC_Os10g21406.1	Photosystem I iron-sulfur center	Increases the Photosynthetic efficiency of plant
TP1431319	GY	*Setaria italica*	scaffold_14	Seita.J014000.1	Photosystem II protein (PSII)-cytochrome b6/f complex	Increases the rate of Electron transport through, which leads to increased rate of photosynthesis, plant growth and ultimately grain yield
TP872087	DM	*Setaria italica*	Scaffold_7	Seita.7G269600.1	GRF zinc finger	Increases plant grain yield
TP760377	GY	*Setaria italica*	Scaffold_14	Seita.J019200.1	Ribosomal protein	Increases grain yield

## Discussion

### Variation for agro-morphological traits

Significant differences among genotypes for most of the traits showed existence of sufficient variability. The five top yielding accessions in both the locations were of Indian origin except one accession, IE 2710 from Malawi. The trend was similar in both the environments and all further high yielding accessions (65) in both the locations were mostly of Indian origin. Similarly, all low yielding accessions (45) in both the environments were mostly of African origin again. The accessions having grain yield greater than the average grain yield of all the accessions were considered to be high yielding and the remaining were considered to be low yielding (S5 Table). The results of our study were in agreement with Dida et al. [9] for plant height and peduncle length. We found that the accessions belonging to African countries were late in flowering, while most of the Indian accessions were early in flowering. Bharathi [32] also found similar results where the accessions of South Asia (<66.1 days) and Central Africa (<64.33 days) flowered early whereas, South African accessions (>77.4 days) flowered late. However, the basal tiller number was equal in both Indian and exotic accessions across the two locations in our study, whereas, Dida et al. [9] reported less number of basal tillers in African *coracana* population than the Indian population(Fig 1A and 1B). This type of variation might be observed for some traits due to the influence of different growing environments. High heritability values observed for most of the traits indicate that the traits under study were least influenced by the environment in their expression. Therefore, association of SNP markers with phenotypic trait values for marker assisted selection would be rewarding in future studies.

Correlation values for most of the traits were in corroboration to Bharathi [32], who reported a significant positive correlation of days to flowering with ear length and length of longest finger, plant height with ear head length and peduncle length, and ear head length with length of longest finger. A positive correlation of plant height with ear head length, panicle exertion and grain yield was also reported by Sivagurunathan [33]. Similarly, significant and positive correlation between days to maturity and days to flowering was reported by Wolie and Dessalegn [34]. However, 20 associations were contradictory at E2 location.

### Principal component and cluster analysis

On the basis of projection of the accessions on the first two principal components, two major groups were detected. The red dots represent Indian accessions whereas blue dots indicate exotic accessions in the biplot (Fig 3A and 3B). There were overlaps of accessions from both these groups which may be due to similarity of trait values of accessions of both groups and mixing of genes of Indian and exotic accessions resulting in similarity among them. Specified that the separation of accessions was based mainly on agro-morphological characters, the accessions of Indian origin Indian in the first group were characterized by thicker culms, more basal tillers, more fingers, large peduncles, wide fingers and high grain yield. Exotic accessions on the other hand in second group were late flowering and late maturing, but had long ears with long fingers. Our results of two groups in the finger millet collection are in agreement with earlier studies on similar accessions [11].

Two-way cluster analysis broadly separated the accessions based on trait variation. The first group A contained accessions with high culm thickness, basal tillers, peduncle length and high grain yield. This group mostly contained all accessions of Indian origin, indicating that Indian accessions can be used as donors for these traits. The accessions in group B were late in maturity and had long fingers and ears (S1a and S1b Fig). Thus, they are potential candidates for introgression of these traits. There were some overlapping of accessions in both the clusters, which could be due to migration of material and gene flow from one region to another. The results are in agreement with Babu et al [11].

### Linkage disequilibrium

Identification of disequilibrium between markers is highly useful since it is prerequisite before conducting association mapping analysis. Since physical map distances between markers were not available, LD was represented by the disequilibrium matrix visualizing the linear arrangement of LD between polymorphic sites, represented by r^2^, and the probability [35,36]. In the present study 17.9% of the marker pairs showed significant LD at r^2^ > 0.05. Appearance of long haplotypic blocks in the present study might be due to the selection pressure imposed by the breeders for desirable traits which resulted in accumulation of favourable genes in most of the finger millet genotypes. Secondly, the recombination frequency in finger millet is less than that of cross pollinated crops, since it is a self pollinated crop.

### Marker-trait association analysis

The marker-trait association with agro-morphological traits clearly showed the importance of SNP markers in deciphering QTLs for various yield component traits in the present study. Since, SNPs occur in the genomes in much higher frequency than SSRs, they provide high density of markers near a locus of interest, evolutionary stable and do not change significantly from generation to generation. The complex polygenic nature of traits and the polyploid genome of finger millet make the use of association mapping a great challenge compared to other studies conducted on crops with lesser genome complexity.

SLST analysis led to the identification of twenty potential and significant SNPs linked to five useful agronomic traits in both the environments through GLM and MLM approaches. An additional covariate of kinship is included in MLM approach for effective regulation of false discovery of associations as compared to GLM, due to better control for population structure and relatedness within genome-wide association studies [37]. Weaker associations were omitted in the present study which had a p-value of > 0.001, as they might give misleading results. Besides, the present study provides an improvement upon single-locus single trait analysis (SLST) through the application of MTMM and MLMM approaches, which have been recently used to identify SSR markers linked to useful agronomic traits in common wheat [6]. Ten of the 53 marker-trait associations were common with those identified through SLST approach. MLMM approach additionally identified 36 unique MTAs, other than 10 common associations to that of SLST. This clearly showed that MLMM approach is more powerful than SLST to overcome the problem of genetic background which decreases the efficiency of QTL detection [7]. It was interesting to observe that the SNP markers identified to be associated with agronomic traits had high heritability, indicating robustness and practical utility of markers in finger millet breeding programmes.

Further, MTMM analysis identified 24 pleiotropic MTAs for both the environments involving QTLs that are associated with eight pairs of correlated traits (out of 15 pairs of correlated traits examined). It can be speculated that the remaining correlations might be due to environmental effect or LD rather than pleiotropy /linkage [6]. These 24 MTAs might be useful for the improvement of correlated traits simultaneously. The use of MTMM could be extended from analyzing pairs of correlated traits to multi-traits. Such experiments have been well studied in beef cattle [38] and humans [39] and may be used in plants in near future.

Epistasis or interactions between non allelic genes is an important factor that affects phenotypic expression of genes and genetic variation in populations [40]. Epistatic loci identification is a key step towards resolving discrepancies between QTL mapping and classical genetic dogma. It also helps in better understanding of the persistence of quantitative genetic variation in populations and designing appropriate marker-assisted breeding strategies for improvement of complex traits [41]. Although most QTL mapping studies reveal little evidence for the presence of epistasis between QTLs [42–49], “genetic background effects” on quantitative traits have been well documented in Drosophila [50], tomato [51], rice [52], soybean [53], maize [54,55] and wheat [6]. These observations suggest that there exist epistatic loci that may affect the expression of genes or QTLs, causing background effects. Our results also indicate that epistatic interactions were important in the genetic control of grain yield and most of its component traits in finger millet.

The three different approaches SLST, MLMM and MTMM identified nine common SNPs associations with agronomic traits. *In silico* analysis showed multiple hits for eight SNPs in the different grass genomes. Five out of these eight SNP markers were found orthologous to candidate genes of *Oryza sativa* and *Setaria italica*, encoding ATP synthase, photosystem I, photosystem II, GRF zinc finger and ribosomal protein, which play an important role in plant growth and grain yield (Table 5). The SNPs TP1431319, TP214730 and TP760377 had A to T transversion, TP1071491 had A to G transition, TP872087 had G to C transition (S6 Table). In tobacco, it was found that repressing the ATP synthase ultimately leads to repression in photosynthetic electron transport, CO_2_ assimilation and plant growth by over-acidification of the thylakoid lumen [56]. It indicates that this gene plays an important role in plant growth and flowering. The SNP, TP872087 was found associated with the gene for GRF zinc finger. It has been reported that plants having increased expression of a nucleic acid sequence encoding a GRF (Growth Regulating Factor) polypeptide lead to increased yield related traits in comparison to control plants (WO2009034188 A1). Another SNP, TP760377 was associated with gene for ribosomal protein S2, which is also associated with increased plant yield by increasing the activity of ribosomal protein in plants or plant parts (WO2011061656 A1). We have identified several cross genome orthologues to the identified SNP markers linked to various agronomic traits in finger millet, that were proximal to several candidate genes that are known to regulate the respective traits in other grass species. However, there are no previous reports on the role of these candidate genes in finger millet. Therefore, these genes require independent validation for their role in finger millet genome. KEGG analysis revealed that the genes encoding ATP synthase, Photosystem I and Photosystem II are predominately involved in photosynthetic and oxidative phosphorylation pathways, which ultimately could play an important role in enhancement of finger millet grain yield.

The SNP markers identified in the present study and their putatively associated candidate genes have been reported for the first time in finger millet. On validation, these novel QTLs may be utilized in future for marker assisted breeding for the development of high yielding varieties of finger millet.

#### Conclusion

Finger millet genotypes used in the study showed wide genetic variation with respect to agro-morphological traits. Indian and exotic finger millet genotypes showed differences in trait values and were clustered separately based on trait values. Marker trait association analysis using three different approaches identified nine common SNPs marker associations for grain yield and its component traits. Five out of nine SNPs were found to have orthologous candidate genes for plant growth and grain yield in related genera through *insilico* analysis. These linked markers can be further used for cloning of the full length genes, fine mapping and ultimately in the marker-assisted breeding programmes for introgression of alleles into locally well adapted genotypes. The lack of complete genome sequence of finger millet is a major bottle neck for developing a molecular marker based breeding programme till date, but the SNP markers generated through next generation sequencing technologies such as genotyping-by-sequencing analysis (GBS) in the present study could provide great leads in this direction. Genome wide association studies for grain yield and its components would help in the identification of loci associated with each trait and subsequently genomic regions could be highlighted for targeted resequencing for marker discovery in finger millet.

## Supporting information

S1 Fig a) Cluster analysis of 202 genotypes on the basis of 14 agro-morphological traits at E1. b) Cluster analysis of 202 genotypes on the basis of 14 agro-morphological traits at E2.
(TIF)

S2 Fig Linkage disequilibrium (LD) plot. (DOCX)

S3 Fig Pathways involved in finger millet grain yield enhancement. (DOCX)

S1 Table Analysis of Variance of 14 quantitative traits evaluated at E1 and E2. (DOCX)

S2 Table Correlation coefficient values for all possible pairs involving 14 traits evaluated at E1 and E2, indicating significance at 0.05 level. Trait-pair showing correlation coefficient value >0.2 were used in multi-trait analysis and are highlighted in bold.
(DOC)

S3 Table Multi marker single trait and single marker multi trait associations across the two environments. (DOC)

S4 Table Epistatic interactions using main effect markers (identified by SLST, MLMM and MTMM) for 13 traits evaluated at E1 and E2 along with their p value. (DOC)

S5 Table List of Indian and exotic finger millet genotypes. (DOC)

S6 Table Nature of substitution in the identified SNP markers. (DOC)
