# Supplementary figures and images for: Genome wide association mapping of agro-morphological traits among a diverse collection of finger millet (Eleusine coracana L.) genotypes using SNP markers
Source: PLoS One. 2018 Aug 9;13(8):e0199444. doi: 10.1371/journal.pone.0199444 (PMC6084814; doi:10.1371/journal.pone.0199444)

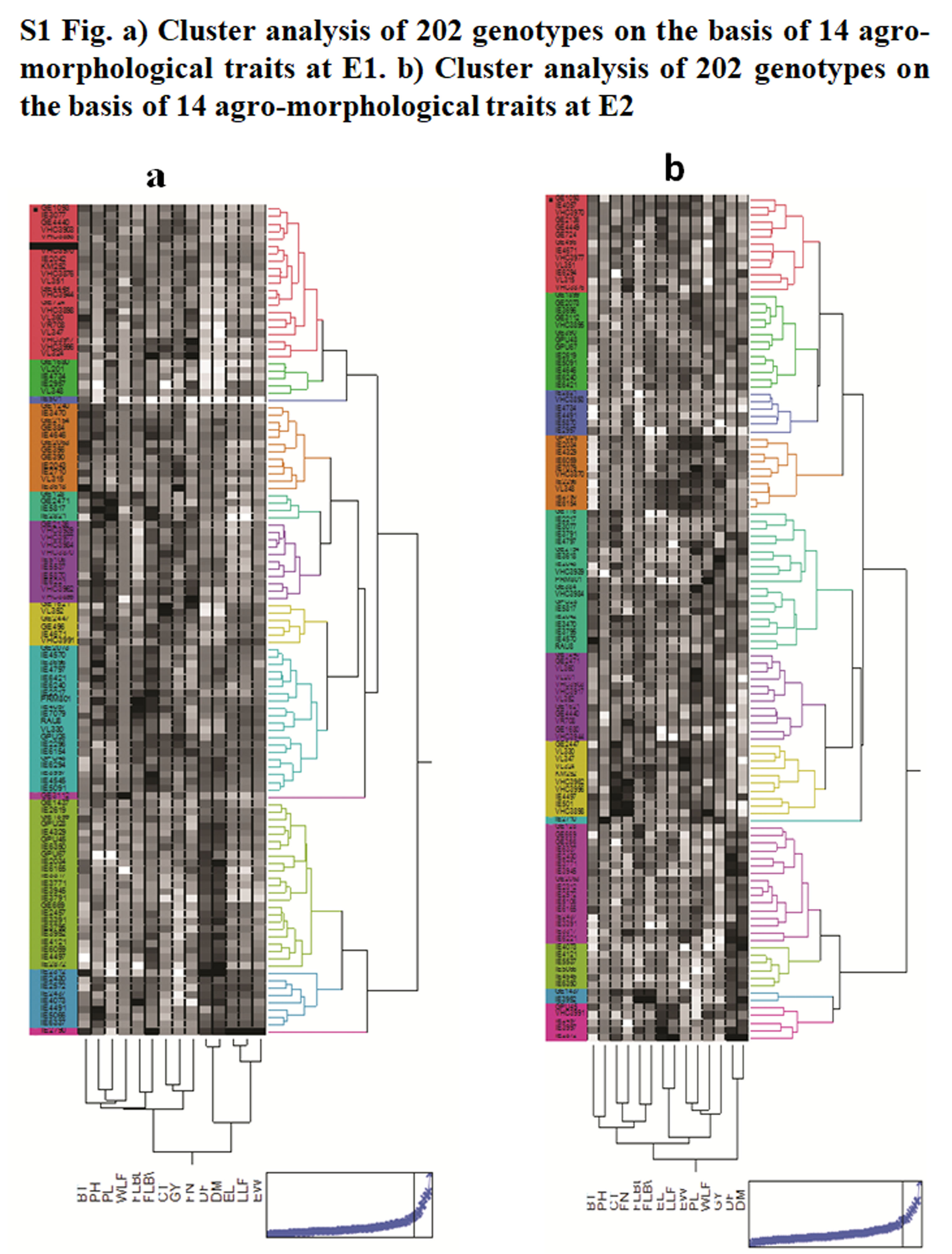

Supplement: S1 Fig — a) Cluster analysis of 202 genotypes on the basis of 14 agro-morphological traits at E1. b) Cluster analysis of 202 genotypes on the basis of 14 agro-morphological traits at E2. (TIF) [file pone.0199444.s001.tif]

**S3 Fig. Pathways involved in finger millet grain yield enhancement.**


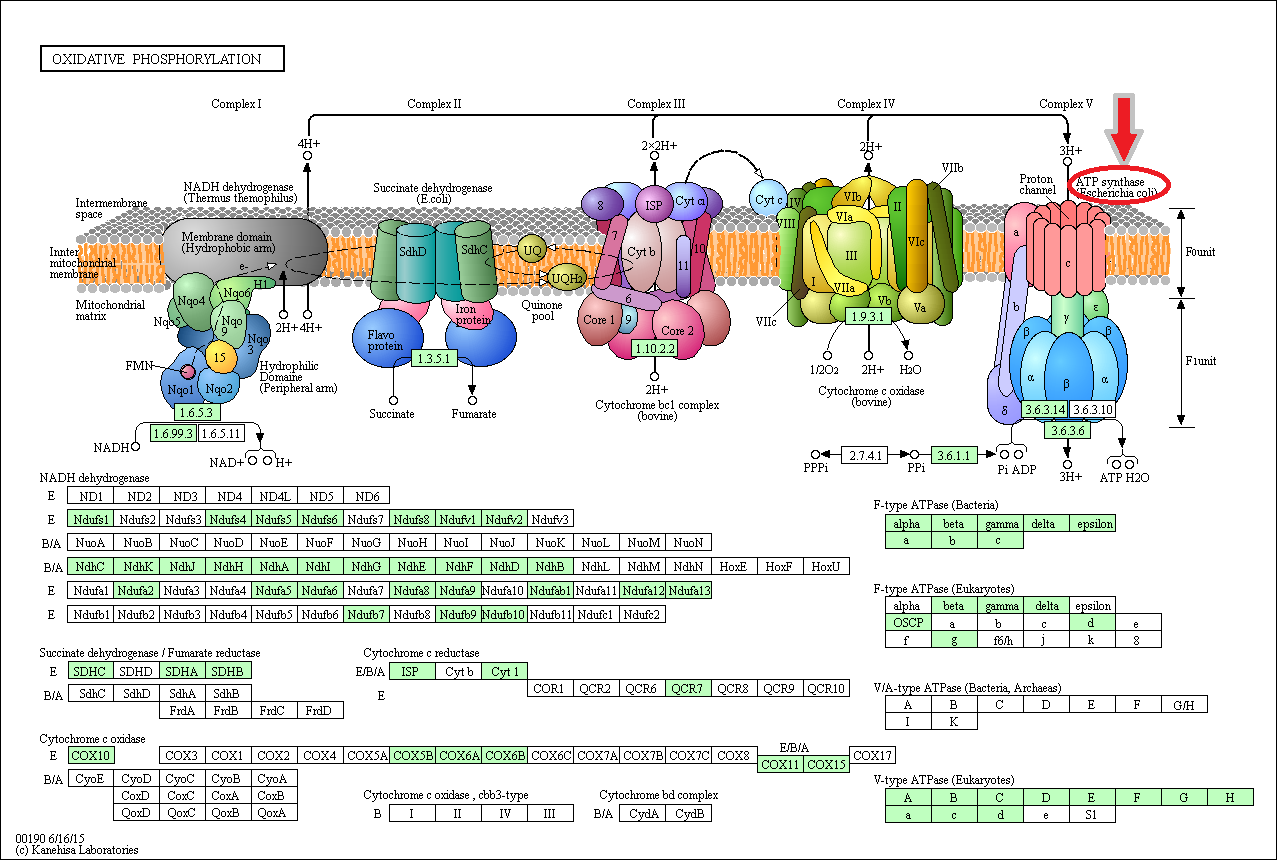

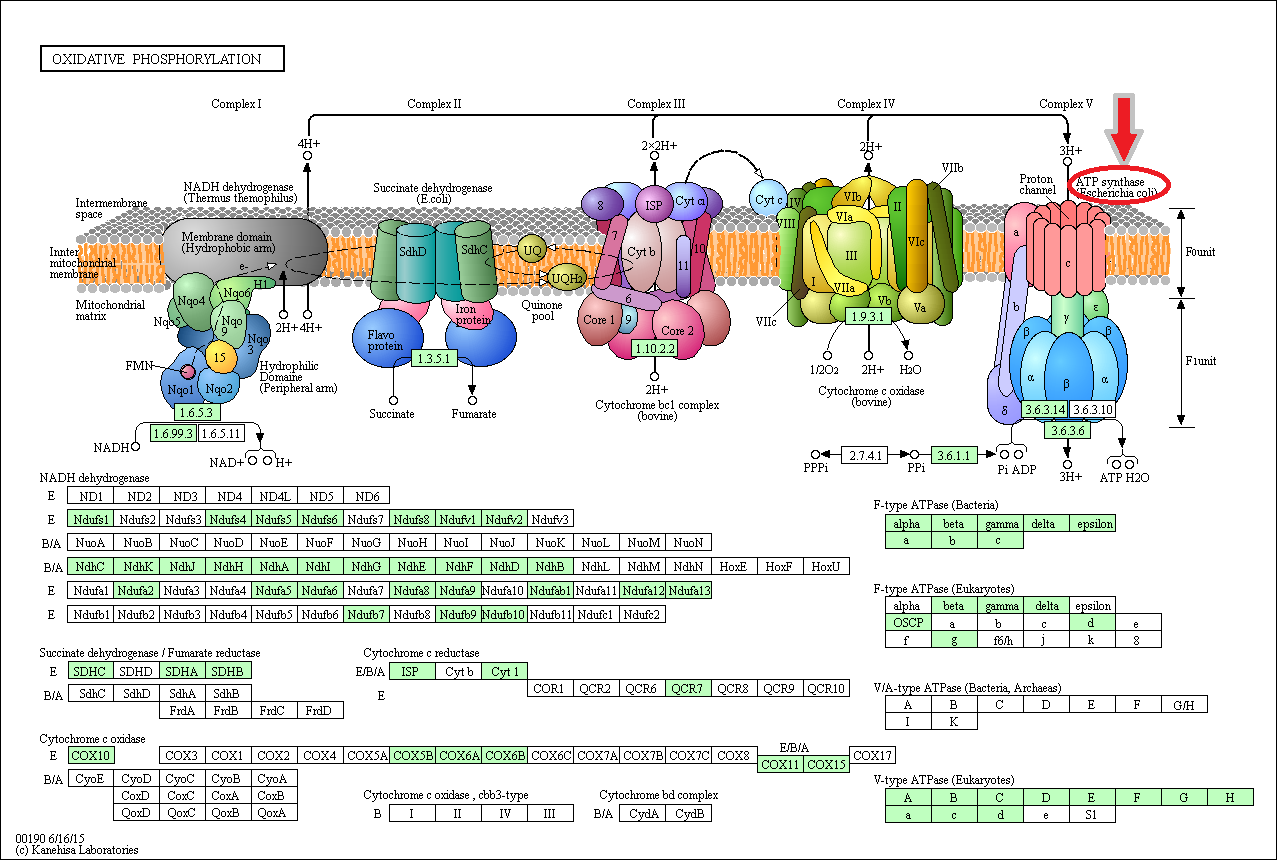

Supplement: S3 Fig — (DOCX) [file pone.0199444.s003.docx]
