# Supplementary material for: Genome wide association mapping of agro-morphological traits among a diverse collection of finger millet (Eleusine coracana L.) genotypes using SNP markers
Source: PLoS One. 2018 Aug 9;13(8):e0199444. doi: 10.1371/journal.pone.0199444 (PMC6084814; doi:10.1371/journal.pone.0199444)
Supplement: S2 Fig — (DOCX) [file pone.0199444.s002.docx]

**S2 Fig. Linkage disequilibrium (LD) plot**

**
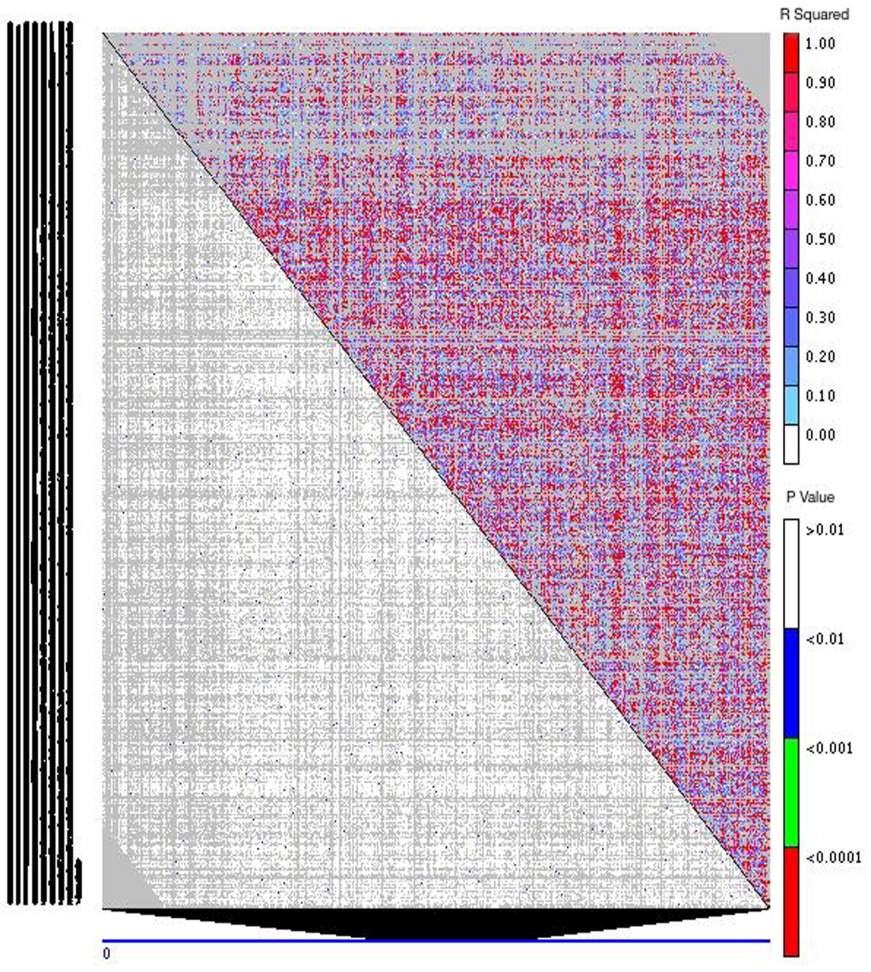
**

**S2 Fig. Linkage disequilibrium (LD) plot.** Each square in the plots represents the level of LD between a pair of sites in a region. The setting graphs are: r2 in the upper right and p- values in the lower left triangles. Their levels of r2 and p-value are presented by colors as illustrated in the right panel. Red coloring indicates strong LD, white indicates weak LD, and green/blue indicates intermediate LD. The left side of the graph contains a text description of the SNP sites. At the bottom of the graph is a display of the position of each site along the chromosome.
