# Supplementary material for: Genome wide association mapping of agro-morphological traits among a diverse collection of finger millet (Eleusine coracana L.) genotypes using SNP markers
Source: PLoS One. 2018 Aug 9;13(8):e0199444. doi: 10.1371/journal.pone.0199444 (PMC6084814; doi:10.1371/journal.pone.0199444)
Supplement: S1 Table — (DOCX) [file pone.0199444.s004.docx]

**S1 Table: ANOVA for 14 quantitative traits evaluated at E1 and E2 respectively**

| **Traits** |  | **Replication** | | **Block (Rep)** | | **Treatment** | | **Error** | |
| --- | --- | --- | --- | --- | --- | --- | --- | --- | --- |
|  | **Df** | **1** | | **36** | | **113** | | **77** | |
|  |  | **E1** | **E2** | **E1** | **E2** | **E1** | **E2** | **E1** | **E2** |
| **CT** |  | 10.145 | 1.463 | 2.267 | 0.311 | 2.4529** | 0.529** | 1.724 | 0.307 |
| **DF** |  | 466.019 | 7.826 | 35.249 | 86.536 | 290.651** | 194.346** | 22.051 | 108.997 |
| **DM** |  | 417.052 | 4.600 | 28.030 | 49.304 | 303.725** | 284.094** | 17.792 | 23.356 |
| **EL** |  | 0.103 | 3.042 | 1.017 | 17.715 | 7.893** | 22.506** | 0.868 | 15.964 |
| **EW** |  | 1.939 | 5.723 | 0.405 | 1.478 | 1.747** | 2.138** | 0.266 | 0.732 |
| **FLBL** |  | 86.695 | 2.256 | 22.462 | 4.954 | 26.378** | 91.317** | 8.102 | 4.537 |
| **FLBW** |  | 0.044 | 1.509 | 0.006 | 0.163 | 0.005** | 0.168 | 0.002 | 0.133 |
| **LLF** |  | 2.240 | 0.936 | 0.662 | 1.997 | 5.957** | 5.798** | 0.501 | 1.352 |
| **FN** |  | 0.678 | 6.672 | 0.755 | 0.736 | 3.125** | 3.987** | 0.609 | 0.525 |
| **BT** |  | 18.802 | 32.303 | 1.161 | 0.742 | 0.960* | 4.859** | 0.745 | 0.938 |
| **PH** |  | 2289.913 | 7966.484 | 203.372 | 4622.879 | 340.917** | 5540.388 | 51.545 | 4852.250 |
| **PL** |  | 24.842 | 39.514 | 8.934 | 5.968 | 17.076** | 55.467** | 3.681 | 6.965 |
| **WLF** |  | 0.122 | 0.050 | 0.036 | 0.055 | 0.047 | 0.388** | 0.042 | 0.061 |
| **GY** |  | 0.020 | 0.006 | 0.006 | 0.001 | 0.017** | 0.018** | 0.003 | 0.000 |
