# Supplementary material for: Genome wide association mapping of agro-morphological traits among a diverse collection of finger millet (Eleusine coracana L.) genotypes using SNP markers
Source: PLoS One. 2018 Aug 9;13(8):e0199444. doi: 10.1371/journal.pone.0199444 (PMC6084814; doi:10.1371/journal.pone.0199444)
Supplement: S2 Table — Trait-pair showing correlation coefficient value >0.2 were used in multi-trait analysis and are highlighted in bold. (DOC) [file pone.0199444.s005.doc]

**S2 Table**

**Correlation coefficient values for all possible pairs involving 14 traits evaluated at E1, indicating significance at 0.05 level. Trait-pair showing correlation coefficient value >0.2 were used in multi-trait analysis and are highlighted in bold.**

**Correlation coefficient values for all possible pairs involving 14 traits evaluated at E2, indicating significance at 0.05 level. Trait-pair showing correlation coefficient value >0.2 were used in multi-trait analysis and are highlighted in bold.**
