# Supplementary material for: Genome wide association mapping of agro-morphological traits among a diverse collection of finger millet (Eleusine coracana L.) genotypes using SNP markers
Source: PLoS One. 2018 Aug 9;13(8):e0199444. doi: 10.1371/journal.pone.0199444 (PMC6084814; doi:10.1371/journal.pone.0199444)
Supplement: S3 Table — (DOC) [file pone.0199444.s006.doc]

**S3 Table: Multi marker single Trait and Single marker m**ulti trait associations across the two environments

| **Environment 1** | | | | **Environment 2** | | | |
| --- | --- | --- | --- | --- | --- | --- | --- |
| **Multiple marker Associated with single traits** | | **Single marker**  **Associated with**  **multiple traits** | | **Multiple marker Associated with single traits** | | **Single Marker associated multiple traits pantnagar** | |
| **Marker** | **Traits** | **Marker** | **Traits** | **Marker** | **Traits** | **Marker** | **Traits** |
| TP1123489 | BT | TP1021485 | EL | TP1056544 | BT | TP1071491 | DF |
| TP1189329 |  |  | EW | TP1121999 |  |  | GY |
| TP1203700 |  |  | LLF | TP1155810 |  |  | DM |
| TP122468 |  | TP103874 | DF | TP118520 |  | TP1205770 | FN |
| TP1246734 |  |  | GY | TP1319246 |  |  | DF |
| TP1259011 |  | TP1071491 | DF | TP133182 |  | TP12628 | DM |
| TP1578768 |  |  | DM | TP1350583 |  |  | BT |
| TP206325 |  |  | GY | TP1408352 |  | TP133182 | DF |
| TP397541 |  | TP1084311 | FN | TP141203 |  |  | DM |
| TP449284 |  |  | WLF | TP1448623 |  |  | DF |
| TP609683 |  | TP1084377 | EL | TP1471284 |  | TP1349783 | EW |
| TP692158 |  |  | EW | TP1472906 |  |  | GY |
| TP692174 |  |  | LLF | TP1487224 |  | TP136828 | PL |
| TP692539 |  | TP1084390 | DF | TP1492633 |  |  | GY |
| TP8116 |  |  | DM | TP1591837 |  | TP1431319 | PH |
| TP845271 |  | TP1084756 | EL | TP245137 |  |  | EL |
| TP900088 |  |  | LLF | TP316443 |  | TP1573416 | LLF |
| TP1003604 | CT | TP1193783 | DF | TP402835 |  |  | DF |
| TP1084719 |  |  | DM | TP481672 |  | TP1600637 | DM |
| TP1251570 |  | TP1242017 | EL | TP482930 |  |  | DF |
| TP1256507 |  |  | EW | TP591112 |  | TP214730 | DM |
| TP1507071 |  |  | LLF | TP595219 |  |  | FN |
| TP203445 |  | TP128765 | DF | TP658615 |  | TP224559 | WLF |
| TP254607 |  |  | DM | TP795215 |  |  | PH |
| TP339430 |  |  | PL | TP821933 |  | TP328617 | WLF |
| TP728508 |  | TP133182 | DF | TP1047214 | CT |  | BT |
| TP80999 |  |  | DM | TP1060609 |  | TP481672 | CT |
| TP940052 |  | TP1431319 | EW | TP1062677 |  |  | FN |
| TP103874 | DF |  | GY | TP107921 |  |  | DM |
| TP1071491 |  | TP1506652 | EL | TP1084306 |  | TP58552 | PH |
| TP1084301 |  |  | EW | TP1084410 |  |  | DF |
| TP1084390 |  |  | LLF | TP1260366 |  | TP692257 | GY |
| TP1193783 |  | TP1513069 | FLBW | TP1340336 |  |  | FLBL |
| TP128765 |  |  | WLF | TP299433 |  | TP692601 | FLBW |
| TP133182 |  | TP1600637 | DF | TP470219 |  |  | PH |
| TP1406447 |  |  | DM | TP481672 |  |  | GY |
| TP1574491 |  | TP212 | EL | TP599331 |  | TP760377 | LLF |
| TP1586020 |  |  | LLF | TP628586 |  |  | DF |
| TP1600637 |  | TP213753 | EW | TP635573 |  | TP774435 | PL |
| TP187612 |  |  | LLF | TP728528 |  |  | DF |
| TP235181 |  | TP258294 | FLBW | TP802503 |  | TP812952 | DM |
| TP262420 |  |  | WLF | TP862562 |  |  | GY |
| TP361263 |  | TP289504 | EL | TP1071491 | DF | TP878687 | DF |
| TP393542 |  |  | EW | TP1131467 |  |  | DF |
| TP414254 |  | TP351642 | EL | TP1217728 |  | TP960312 | PL |
| TP414432 |  |  | EW | TP12628 |  |  | GY |
| TP423850 |  | TP411070 | EL | TP133182 |  |  | DF |
| TP547232 |  |  | EW | TP1349783 |  |  | DM |
| TP58552 |  |  | LLF | TP1357157 |  |  | |
| TP627932 |  | TP414254 | DF | TP1512686 |  |  |  |
| TP682023 |  |  | DM | TP1540383 |  |  |  |
| TP859171 |  | TP414432 | DF | TP1600637 |  |  |  |
| TP872087 |  |  | DM | TP214730 |  |  |  |
| TP979901 |  | TP423850 | DF | TP462510 |  |  |  |
| TP1071491 | DM |  | GY | TP471968 |  |  |  |
| TP1084390 |  |  | PL | TP525918 |  |  |  |
| TP1193783 |  | TP525607 | GY | TP692257 |  |  |  |
| TP128765 |  |  | WLF | TP701554 |  |  |  |
| TP1331459 |  | TP547232 | DF | TP774435 |  |  |  |
| TP133182 |  |  | DM | TP804096 |  |  |  |
| TP1381676 |  |  | PL | TP812952 |  |  |  |
| TP1581043 |  | TP548665 | EL | TP878687 |  |  |  |
| TP1600637 |  |  | EW | TP891789 |  |  |  |
| TP214730 |  |  | LLF | TP960312 |  |  |  |
| TP414254 |  | TP58758 | EL | TP1084442 | DM |  |  |
| TP414432 |  |  | EW | TP1084844 |  |  |  |
| TP547232 |  | TP627932 | DF | TP1205770 |  |  |  |
| TP627932 |  |  | DM | TP12628 |  |  |  |
| TP692888 |  | TP682023 | DF | TP133182 |  |  |  |
| TP777692 |  |  | PL | TP1355538 |  |  |  |
| TP872087 |  | TP692075 | EL | TP1381676 |  |  |  |
| TP1021485 | EL |  | LLF | TP1600637 |  |  |  |
| TP1084377 |  | TP692158 | BT | TP214730 |  |  |  |
| TP1084756 |  |  | PH | TP414254 |  |  |  |
| TP1147236 |  | TP731912 | EL | TP58552 |  |  |  |
| TP1242017 |  |  | EW | TP626604 |  |  |  |
| TP1277572 |  |  | LLF | TP627932 |  |  |  |
| TP1332862 |  | TP794211 | EW | TP675608 |  |  |  |
| TP1506652 |  |  | LLF | TP812952 |  |  |  |
| TP212 |  | TP859171 | DF | TP872087 |  |  |  |
| TP251357 |  |  | PL | TP920450 |  |  |  |
| TP289504 |  | TP872087 | DF | TP960312 |  |  |  |
| TP351642 |  |  | DM | TP979901 |  |  |  |
| TP411070 |  | TP878687 | EL | TP1086452 | EL |  |  |
| TP548665 |  |  | EW | TP1086502 |  |  |  |
| TP58758 |  |  | GY | TP1377112 |  |  |  |
| TP692075 |  |  | LLF | TP1573416 |  |  |  |
| TP731912 |  | TP915260 | FN | TP1587737 |  |  |  |
| TP746525 |  |  | PH | TP335802 |  |  |  |
| TP878687 |  | TP940052 | CT | TP351642 |  |  |  |
| TP9600 |  |  | GY | TP863610 |  |  |  |
| TP982826 |  | TP9600 | EL | TP938485 |  |  |  |
| TP1021485 | EW |  | FN | TP1012638 | EW |  |  |
| TP1084377 |  |  | LLF | TP1085271 |  |  |  |
| TP1084736 |  | TP982826 | EL | TP1288966 |  |  |  |
| TP1242017 |  |  | EW | TP1349783 |  |  |  |
| TP1431319 |  |  | LLF | TP1387646 |  |  |  |
| TP1506652 |  |  | | TP1548988 |  |  |  |
| TP1574043 |  |  |  | TP1562177 |  |  |  |
| TP213753 |  |  |  | TP1577550 |  |  |  |
| TP289504 |  |  |  | TP1589514 |  |  |  |
| TP351642 |  |  |  | TP661794 |  |  |  |
| TP411070 |  |  |  | TP936915 |  |  |  |
| TP548665 |  |  |  | TP1084632 | FLBL |  |  |
| TP557333 |  |  |  | TP1085678 |  |  |  |
| TP58758 |  |  |  | TP538536 |  |  |  |
| TP731912 |  |  |  | TP572397 |  |  |  |
| TP731945 |  |  |  | TP641195 |  |  |  |
| TP76640 |  |  |  | TP686339 |  |  |  |
| TP794211 |  |  |  | TP692601 |  |  |  |
| TP808535 |  |  |  | TP754931 |  |  |  |
| TP878687 |  |  |  | TP807139 |  |  |  |
| TP982826 |  |  |  | TP8087 |  |  |  |
| TP1082962 | FLBL |  |  | TP976267 |  |  |  |
| TP1200526 |  |  |  | TP1053372 | FLBW |  |  |
| TP1271003 |  |  |  | TP1084311 |  |  |  |
| TP1517789 |  |  |  | TP1085137 |  |  |  |
| TP1518966 |  |  |  | TP1085937 |  |  |  |
| TP641195 |  |  |  | TP1193783 |  |  |  |
| TP849482 |  |  |  | TP1308290 |  |  |  |
| TP888984 |  |  |  | TP1381285 |  |  |  |
| TP990914 |  |  |  | TP1475736 |  |  |  |
| TP1060609 | FLBW |  |  | TP1525039 |  |  |  |
| TP1084317 |  |  |  | TP1587362 |  |  |  |
| TP1084327 |  |  |  | TP193202 |  |  |  |
| TP1084335 |  |  |  | TP253281 |  |  |  |
| TP1084353 |  |  |  | TP312668 |  |  |  |
| TP1084388 |  |  |  | TP39266 |  |  |  |
| TP1084402 |  |  |  | TP470899 |  |  |  |
| TP1084630 |  |  |  | TP58758 |  |  |  |
| TP1087185 |  |  |  | TP620834 |  |  |  |
| TP110014 |  |  |  | TP692074 |  |  |  |
| TP1127171 |  |  |  | TP692100 |  |  |  |
| TP1344258 |  |  |  | TP692440 |  |  |  |
| TP1513069 |  |  |  | TP692601 |  |  |  |
| TP209450 |  |  |  | TP1055730 | FN |  |  |
| TP233637 |  |  |  | TP1093957 |  |  |  |
| TP258294 |  |  |  | TP1205770 |  |  |  |
| TP520254 |  |  |  | TP1482654 |  |  |  |
| TP600436 |  |  |  | TP18538 |  |  |  |
| TP655094 |  |  |  | TP216529 |  |  |  |
| TP734421 |  |  |  | TP224559 |  |  |  |
| TP1051423 | FN |  |  | TP257739 |  |  |  |
| TP1084311 |  |  |  | TP326115 |  |  |  |
| TP1247539 |  |  |  | TP37021 |  |  |  |
| TP1367644 |  |  |  | TP420675 |  |  |  |
| TP1378774 |  |  |  | TP481672 |  |  |  |
| TP425601 |  |  |  | TP63527 |  |  |  |
| TP658615 |  |  |  | TP692177 |  |  |  |
| TP891789 |  |  |  | TP692179 |  |  |  |
| TP915260 |  |  |  | TP693337 |  |  |  |
| TP9600 |  |  |  | TP819696 |  |  |  |
| TP103874 | GY |  |  | TP821806 |  |  |  |
| TP1071491 |  |  |  | TP840665 |  |  |  |
| TP1310091 |  |  |  | TP1071491 | GY |  |  |
| TP1431319 |  |  |  | TP1084631 |  |  |  |
| TP1510146 |  |  |  | TP1310091 |  |  |  |
| TP186343 |  |  |  | TP136828 |  |  |  |
| TP28411 |  |  |  | TP1431319 |  |  |  |
| TP400108 |  |  |  | TP1439022 |  |  |  |
| TP423850 |  |  |  | TP1510146 |  |  |  |
| TP498100 |  |  |  | TP227742 |  |  |  |
| TP525607 |  |  |  | TP28411 |  |  |  |
| TP554491 |  |  |  | TP64954 |  |  |  |
| TP60652 |  |  |  | TP673880 |  |  |  |
| TP64954 |  |  |  | TP692174 |  |  |  |
| TP691558 |  |  |  | TP692257 |  |  |  |
| TP760377 |  |  |  | TP735980 |  |  |  |
| TP878687 |  |  |  | TP752454 |  |  |  |
| TP940052 |  |  |  | TP760377 |  |  |  |
| TP1021485 | LLF |  |  | TP812952 |  |  |  |
| TP1084377 |  |  |  | TP878687 |  |  |  |
| TP1084756 |  |  |  | TP982595 |  |  |  |
| TP1102123 |  |  |  | TP1084541 | LLF |  |  |
| TP1190218 |  |  |  | TP1084736 |  |  |  |
| TP1242017 |  |  |  | TP118310 |  |  |  |
| TP1506652 |  |  |  | TP1283158 |  |  |  |
| TP1579530 |  |  |  | TP1573416 |  |  |  |
| TP212 |  |  |  | TP284960 |  |  |  |
| TP213753 |  |  |  | TP564118 |  |  |  |
| TP411070 |  |  |  | TP692198 |  |  |  |
| TP548665 |  |  |  | TP760377 |  |  |  |
| TP692075 |  |  |  | TP893562 |  |  |  |
| TP731912 |  |  |  | TP900088 |  |  |  |
| TP794211 |  |  |  | TP1012762 | PH |  |  |
| TP878687 |  |  |  | TP1016851 |  |  |  |
| TP9600 |  |  |  | TP10477 |  |  |  |
| TP982826 |  |  |  | TP1084543 |  |  |  |
| TP1084546 | PH |  |  | TP1086427 |  |  |  |
| TP1121673 |  |  |  | TP1128733 |  |  |  |
| TP1157411 |  |  |  | TP1155963 |  |  |  |
| TP1333691 |  |  |  | TP1176252 |  |  |  |
| TP1510943 |  |  |  | TP125415 |  |  |  |
| TP175958 |  |  |  | TP1333808 |  |  |  |
| TP376664 |  |  |  | TP1360524 |  |  |  |
| TP461577 |  |  |  | TP1377925 |  |  |  |
| TP540740 |  |  |  | TP1431319 |  |  |  |
| TP577576 |  |  |  | TP1511869 |  |  |  |
| TP692123 |  |  |  | TP1515384 |  |  |  |
| TP692158 |  |  |  | TP1539767 |  |  |  |
| TP692389 |  |  |  | TP208161 |  |  |  |
| TP693262 |  |  |  | TP305637 |  |  |  |
| TP797646 |  |  |  | TP324493 |  |  |  |
| TP836154 |  |  |  | TP328617 |  |  |  |
| TP915260 |  |  |  | TP344406 |  |  |  |
| TP945533 |  |  |  | TP58552 |  |  |  |
| TP965793 |  |  |  | TP639331 |  |  |  |
| TP991487 |  |  |  | TP646470 |  |  |  |
| TP1084319 | PL |  |  | TP685730 |  |  |  |
| TP1136962 |  |  |  | TP692176 |  |  |  |
| TP128765 |  |  |  | TP692601 |  |  |  |
| TP1313841 |  |  |  | TP707080 |  |  |  |
| TP1331856 |  |  |  | TP717873 |  |  |  |
| TP1573416 |  |  |  | TP78209 |  |  |  |
| TP196545 |  |  |  | TP8158 |  |  |  |
| TP224559 |  |  |  | TP820116 |  |  |  |
| TP256548 |  |  |  | TP869459 |  |  |  |
| TP423850 |  |  |  | TP1043416 | PL |  |  |
| TP547232 |  |  |  | TP1084390 |  |  |  |
| TP682023 |  |  |  | TP1084761 |  |  |  |
| TP692256 |  |  |  | TP1321990 |  |  |  |
| TP701554 |  |  |  | TP136828 |  |  |  |
| TP817431 |  |  |  | TP1592468 |  |  |  |
| TP859171 |  |  |  | TP216990 |  |  |  |
| TP866426 |  |  |  | TP291893 |  |  |  |
| TP1014438 | WLF |  |  | TP459329 |  |  |  |
| TP1084310 |  |  |  | TP726556 |  |  |  |
| TP1084311 |  |  |  | TP774435 |  |  |  |
| TP1084324 |  |  |  | TP815112 |  |  |  |
| TP1084333 |  |  |  | TP1031693 | WLF |  |  |
| TP1084442 |  |  |  | TP1085276 |  |  |  |
| TP1084459 |  |  |  | TP1263740 |  |  |  |
| TP1086526 |  |  |  | TP1504664 |  |  |  |
| TP1087451 |  |  |  | TP1579530 |  |  |  |
| TP1212827 |  |  |  | TP224559 |  |  |  |
| TP1224339 |  |  |  | TP278177 |  |  |  |
| TP123407 |  |  |  | TP328617 |  |  |  |
| TP123798 |  |  |  | TP337598 |  |  |  |
| TP1513069 |  |  |  | TP417296 |  |  |  |
| TP1557183 |  |  |  | TP777692 |  |  |  |
| TP195590 |  |  |  | TP818865 |  |  |  |
| TP236189 |  |  |  |  | |  |  |
| TP258294 |  |  |  |  |  |  |  |
| TP413002 |  |  |  |  |  |  |  |
| TP450 |  |  |  |  |  |  |  |
| TP480800 |  |  |  |  |  |  |  |
| TP525607 |  |  |  |  |  |  |  |
|  |  |  |  |  |  |  |  |
| TP527959 |  |  | |  | |  |  |
| TP664933 |  |  |  |  |  |  |  |
| TP691858 |  |  |  |  |  |  |  |
| TP692072 |  |  |  |  |  |  |  |
| TP692084 |  |  |  |  |  |  |  |
| TP692099 |  |  |  |  |  |  |  |
| TP692110 |  |  |  |  |  |  |  |
| TP692136 |  |  |  |  |  |  |  |
| TP692258 |  |  |  |  |  |  |  |
| TP728527 |  |  |  |  |  |  |  |
| TP738302 |  |  |  |  |  |  |  |
| TP780678 |  |  |  |  |  |  |  |
| TP913036 |  |  |  |  |  |  |  |
| TP937107 |  |  |  |  |  |  |  |
