# Supplementary material for: Genome wide association mapping of agro-morphological traits among a diverse collection of finger millet (Eleusine coracana L.) genotypes using SNP markers
Source: PLoS One. 2018 Aug 9;13(8):e0199444. doi: 10.1371/journal.pone.0199444 (PMC6084814; doi:10.1371/journal.pone.0199444)
Supplement: S4 Table — (DOC) [file pone.0199444.s007.doc]

**S4 Table: Epistatic interactions using main effect markers (identified by SLST, MLMM and MTMM) for 13 traits evaluated at E1 along with their p value**

| **Trait** | **Marker1** | **Marker2** | **P value <0.001** |
| --- | --- | --- | --- |
| **BT** | TP692180 | TP1431319 | 6.83E-04 |
|  | TP107921 | TP18663 | 9.67E-04 |
| **CT** | TP404137 | TP40780 | 1.98E-04 |
|  | TP752454 | TP40780 | 2.01E-04 |
|  | TP9600 | TP40780 | 2.04E-04 |
|  | TP1431319 | TP40780 | 2.23E-04 |
|  | TP1439022 | TP40780 | 2.25E-04 |
|  | TP741818 | TP40780 | 3.57E-04 |
|  | TP1521889 | TP40780 | 3.60E-04 |
|  | TP107921 | TP40780 | 4.05E-04 |
|  | TP1084458 | TP40780 | 4.12E-04 |
|  | TP290701 | TP40780 | 4.43E-04 |
|  | TP1071491 | TP40780 | 4.62E-04 |
|  | TP18663 | TP40780 | 4.71E-04 |
| **DF** | TP979901 | TP1355465 | 1.90E-04 |
|  | TP692180 | TP1565065 | 7.90E-04 |
| **DM** | TP979901 | TP1355465 | 5.81E-04 |
|  | TP1084458 | TP698175 | 9.50E-04 |
| **EL** | TP959938 | TP692180 | 1.01E-04 |
|  | TP45157 | TP760377 | 1.11E-04 |
|  | TP959938 | TP498100 | 2.04E-04 |
|  | TP692180 | TP45157 | 3.19E-04 |
|  | TP959938 | TP962050 | 4.05E-04 |
|  | TP959938 | TP45157 | 4.31E-04 |
|  | TP959938 | TP404137 | 4.97E-04 |
|  | TP45157 | TP498100 | 5.33E-04 |
|  | TP959938 | TP1565065 | 6.23E-04 |
|  | TP959938 | TP698175 | 6.85E-04 |
|  | TP959938 | TP1578768 | 6.86E-04 |
|  | TP959938 | TP1439022 | 7.55E-04 |
|  | TP959938 | TP1086421 | 8.05E-04 |
|  | TP959938 | TP12628 | 8.87E-04 |
| **EW** | TP959938 | TP1565065 | 1.58E-04 |
|  | TP1086421 | TP1581043 | 1.63E-04 |
|  | TP959938 | TP404137 | 1.69E-04 |
|  | TP959938 | TP1578768 | 1.81E-04 |
|  | TP959938 | TP12628 | 1.84E-04 |
|  | TP959938 | TP962050 | 1.85E-04 |
|  | TP959938 | TP1086421 | 1.85E-04 |
|  | TP959938 | TP1439022 | 1.95E-04 |
|  | TP959938 | TP979901 | 1.96E-04 |
|  | TP959938 | TP1355465 | 2.02E-04 |
|  | TP959938 | TP752454 | 2.05E-04 |
|  | TP959938 | TP698175 | 2.09E-04 |
|  | TP959938 | TP1414942 | 2.34E-04 |
|  | TP959938 | TP1085280 | 2.49E-04 |
|  | TP959938 | TP595219 | 2.74E-04 |
|  | TP959938 | TP107921 | 3.34E-04 |
|  | TP45157 | TP760377 | 3.58E-04 |
| **FLBL** | TP1242062 | TP979901 | 3.16E-04 |
|  | TP473053 | TP1510146 | 8.90E-04 |
| **FN** | TP498100 | TP548015 | 2.38E-04 |
| **LLF** | TP692180 | TP45157 | 1.10E-04 |
|  | TP959938 | TP498100 | 1.92E-04 |
|  | TP959938 | TP45157 | 2.14E-04 |
|  | TP1329370 | TP1377207 | 2.74E-04 |
|  | TP959938 | TP404137 | 3.62E-04 |
|  | TP959938 | TP698175 | 3.69E-04 |
|  | TP959938 | TP962050 | 3.71E-04 |
|  | TP959938 | TP1086421 | 4.32E-04 |
|  | TP959938 | TP1578768 | 4.43E-04 |
|  | TP45157 | TP498100 | 4.55E-04 |
|  | TP959938 | TP1439022 | 4.75E-04 |
|  | TP959938 | TP752454 | 4.95E-04 |
|  | TP959938 | TP1565065 | 5.00E-04 |
| **PL** | TP1242062 | TP979901 | 3.83E-04 |
|  | TP1242062 | TP290701 | 7.79E-04 |

**Epistatic interactions using main effect markers (identified by SLST, MLMM and MTMM) for 8 traits evaluated at E2 along with their p value**

| **Trait** | **Marker1** | **Marker2** | **P value<0.001** |
| --- | --- | --- | --- |
| **DF** | TP214730 | TP1355465 | 5.72E-04 |
|  | TP1578768 | TP103874 | 6.85E-04 |
| **DM** | TP979901 | TP1355465 | 4.93E-04 |
|  | TP1578768 | TP698175 | 5.11E-04 |
| **EL** | TP1521889 | TP1377207 | 3.15E-04 |
| **EW** | TP1578768 | TP1003465 | 7.22E-04 |
|  | TP1242062 | TP1003465 | 9.07E-04 |
|  | TP214730 | TP103874 | 9.86E-04 |
| **FLBL** | TP214730 | TP1565065 | 4.13E-04 |
|  | TP1001349 | TP486645 | 5.06E-04 |
| **LLF** | TP979901 | TP1431319 | 2.98E-04 |
|  | TP1414942 | TP1086421 | 3.70E-04 |
|  | TP1431319 | TP595219 | 8.91E-04 |
| **PL** | TP698175 | TP595219 | 5.75E-04 |
