# Supplementary material for: Genome wide association mapping of agro-morphological traits among a diverse collection of finger millet (Eleusine coracana L.) genotypes using SNP markers
Source: PLoS One. 2018 Aug 9;13(8):e0199444. doi: 10.1371/journal.pone.0199444 (PMC6084814; doi:10.1371/journal.pone.0199444)
Supplement: S5 Table — (DOC) [file pone.0199444.s008.doc]

**S5 Table: List of Indian and exotic genotypes**

| **S.No.** | **Indian Genotypes** | **Exotic Genotypes** |
| --- | --- | --- |
|  | GE1093 | GE669 |
|  | GE116 | IE2430 |
|  | GE1240 | IE2437 |
|  | GE128 | IE2457 |
|  | GE1437 | IE2572 |
|  | GE1621 | IE2619 |
|  | GE1680 | IE2710 |
|  | GE1899 | IE2790 |
|  | GE2063 | IE2821 |
|  | GE2136 | IE2872 |
|  | GE2154 | IE2957 |
|  | GE2447 | IE3317 |
|  | GE2471 | IE3391 |
|  | GE356 | IE3696 |
|  | GE384 | IE3771 |
|  | GE390 | IE3791 |
|  | GE4440 | IE3795 |
|  | GE4449 | IE3945 |
|  | GE496 | IE3952 |
|  | GE724 | IE4057 |
|  | GPU26 | IE4073 |
|  | GPU28 | IE4121 |
|  | GPU45 | IE4329 |
|  | GPU48 | IE4491 |
|  | GPU67 | IE4497 |
|  | IE2034 | IE4545 |
|  | IE2042 | IE4570 |
|  | IE2043 | IE4646 |
|  | IE2217 | IE4797 |
|  | IE2296 | IE5066 |
|  | IE2312 | IE5091 |
|  | IE3077 | IE5106 |
|  | IE3470 | IE5537 |
|  | IE3997 | IE5817 |
|  | IE4671 | IE5870 |
|  | IE4734 | IE6059 |
|  | IE501 | IE6154 |
|  | KM252 | IE6165 |
|  | PRM801 | IE6221 |
|  | RAU8 | IE6240 |
|  | VHC3870 | IE6294 |
|  | VHC3876 | IE6337 |
|  | VHC3893 | IE6350 |
|  | VHC3895 | IE6421 |
|  | VHC3898 | IE7079 |
|  | VHC3903 |  |
|  | VHC3917 |  |
|  | VHC3939 |  |
|  | VHC3944 |  |
|  | VHC3962 |  |
|  | VHC3970 |  |
|  | VHC3977 |  |
|  | VHC3984 |  |
|  | VHC3991 |  |
|  | VHC3996 |  |
|  | VL201 |  |
|  | VL315 |  |
|  | VL324 |  |
|  | VL330 |  |
|  | VL347 |  |
|  | VL348 |  |
|  | VL351 |  |
|  | VL352 |  |
|  | VL360 |  |
|  | VR708 |  |
