# Supplementary material for: Genome wide association mapping of agro-morphological traits among a diverse collection of finger millet (Eleusine coracana L.) genotypes using SNP markers
Source: PLoS One. 2018 Aug 9;13(8):e0199444. doi: 10.1371/journal.pone.0199444 (PMC6084814; doi:10.1371/journal.pone.0199444)
Supplement: S6 Table — (DOC) [file pone.0199444.s009.doc]

**S6 Table: Nature of substitution in the identified SNP markers**

| **Marker** | **SNP** | **Nature of substitution** |
| --- | --- | --- |
| TP1071491 | A - G | Transition |
| TP133182 | C - T | Transition |
| TP214730 | A - T | Transversion |
| TP872087 | G – C | Transversion |
| TP1310091 | G - T | Transversion |
| TP1431319 | A - T | Transversion |
| TP28411 | A - T | Transversion |
| TP760377 | A - T | Transversion |
| TP878687 | G - T | Transversion |
